# Prostate cancer research: tools, cell types, and molecular targets

**DOI:** 10.3389/fonc.2024.1321694

**Published:** 2024-03-26

**Authors:** Alvin Y. Liu

**Affiliations:** Department of Urology, Institute of Stem Cell and Regenerative Medicine, University of Washington, Seattle, WA, United States

**Keywords:** cancer differentiation, cancer cell reprogramming, stem cell transcription factors, stromal PENK, lineage relationship, AGR2 immunotherapy, differentiation therapy

## Abstract

Multiple cancer cell types are found in prostate tumors. They are either luminal-like adenocarcinoma or less luminal-like and more stem-like non-adenocarcinoma and small cell carcinoma. These types are lineage related through differentiation. Loss of cancer differentiation from luminal-like to stem-like is mediated by the activation of stem cell transcription factors (scTF) such as LIN28A, NANOG, POU5F1 and SOX2. scTF expression leads to down-regulation of β2-microglobulin (B2M). Thus, cancer cells can change from the $\text{scT}\tilde{\text{F}}\text{B}2{\text{M}}^{\mathrm{hi}}$ phenotype of differentiated to that of $\text{scT}\underset{˙}{\text{F}}\text{B}2{\text{M}}^{\mathrm{lo}}$ of dedifferentiated in the disease course. In development, epithelial cell differentiation is induced by stromal signaling and cell contact. One of the stromal factors specific to prostate encodes proenkephalin (PENK). PENK can down-regulate scTF and up-regulate B2M in stem-like small cell carcinoma LuCaP 145.1 cells indicative of exit from the stem state and differentiation. In fact, prostate cancer cells can be made to undergo dedifferentiation or reprogramming by scTF transfection and then to differentiate by PENK transfection. Therapies need to be designed for treating the different cancer cell types. Extracellular anterior gradient 2 (eAGR2) is an adenocarcinoma antigen associated with cancer differentiation that can be targeted by antibodies to lyse tumor cells with immune system components. eAGR2 is specific to cancer as normal cells express only the intracellular form (iAGR2). For AGR2-negative stem-like cancer cells, factors like PENK that can target scTF could be effective in differentiation therapy.

## 1 Differentiated and undifferentiated cancer

By pathology, prostate tumors appear glandular, aglandular, or nonglandular, and histologically unorganized. The Gleason system imparts a numerical value to tumor histology ranging from pattern 3 (G3) showing glandular differentiation, to pattern 4 (G4) showing less glandular differentiation, to pattern 5 (G5) showing no differentiation (1). In large patient cohorts, Gleason scores (GS, sum of two predominant patterns) characterize 46% as GS3 + 3, 41% as GS3 + 4, 11% as GS4 + 3, and 2% as GS≥4 + 4 (2). Thus, most tumors first diagnosed are differentiated, but could become less differentiated over time. Disease upgrading during active surveillance supports this conjecture. On average, 5 years after initial diagnosis, treatment was administered to surveillance patients because of an increase from G3 to G4 (3). What triggers loss of differentiation or dedifferentiation over the disease course remains unclear. Dedifferentiation, as indicated by Gleason upgrading, is correlated with poor outcome (4).

For multicellular organs like the prostate, cell–cell interaction maintains proper differentiation and tissue integrity (5). To investigate this functional aspect, a means to isolate the various component cell types is required so that they could then be combined in certain pairings much like embryonic tissue recombination carried out in the past (6). For the prostate, stromal mesenchyme cells control epithelial differentiation. Defect in this process leads to diseases such as dysplasia, hyperplasia, and neoplasia (6, 7). In cancer, gene expression differences are found not only between luminal and cancer epithelial cells (8) but also between stromal and cancer-associated stromal cells (9). Understanding prostate cancer differentiation, and identifying the genes involved will likely lead to more effective intervention at different stages of the disease.

### 1.1 Prostate cell types by CD staining and transcriptomes

**Figure 1:**
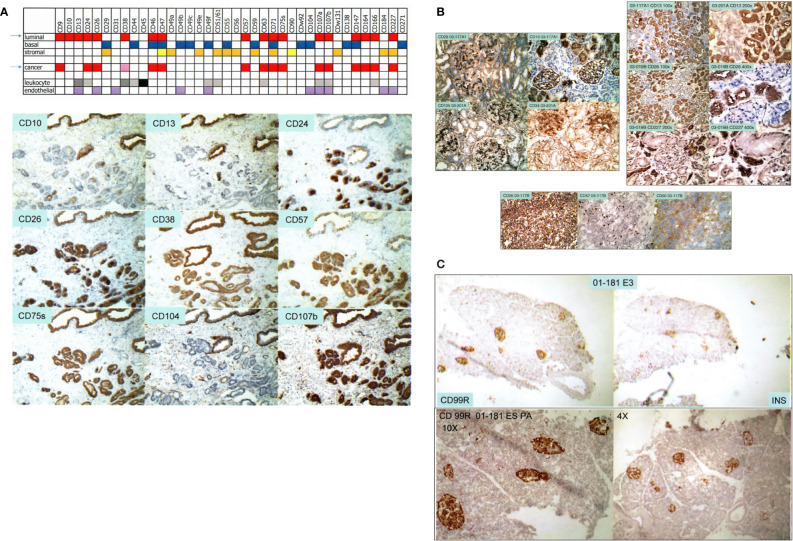
Cell-type CD pattern. **(A)** Top: CD antigens present in the different cell types of the prostate are indicated by color fill-ins. A paler hue denotes lower expression. The arrows point to the close similarity between the CD phenotypes of G3 cancer cells and luminal cells. Bottom: serial sections of one tissue specimen show staining of the CD antibodies indicated. Benign glands are at the top, and tumor glands are at the bottom. **(B)** Shown are reactivities of the CD antibodies on renal cells indicated in the photomicrographs. Positivity is scored by brown staining. The tissue specimens are tagged by an alphanumeric identifier. The left panel shows staining pattern of glomeruli, and the right panel shows that of tubular cells. A renal cell carcinoma (RCC) specimen is stained for CD26, CD57, and CD90. **(C)** The pancreatic islet cells in serially sectioned specimen 01-181E3 are positive for CD99R and insulin (INS). The bottom photomicrographs show CD99R staining of other areas of the tissue section at 10× and 4× magnification.

We used over 200 commercially available antibodies to cluster designation (CD) cell surface antigens to visualize cell types of the prostate in normal/benign vs. cancer. **Figure 1A** shows the CD signatures of luminal, basal, stromal, and cancer cells, plus those of endothelial and leukocytic cells. Cancer cells are like luminal cells except for absent CD10 and CD13, lower CD38, and higher CD24 (10, 11). Cancer-associated stromal cells express a higher level of CD90, in particular, a secreted variant CD90v (12). This characterization allowed us to employ appropriate dye-conjugated CD antibodies to isolate cell populations by flow cytometry: CD26 luminal, CD104 basal, CD49a stromal, CD31 endothelial, CD26 cancer, and CD90 cancer-associated stromal (8, 9, 13). We also used CD phenotyping to visualize cells of the bladder: CD9 urothelial, CD13 stromal of lamina propria, CD104 basal urothelial, CD9 urothelial cancer, and CD13 cancer-associated stromal (14). The applicability of this analytic tool to any organ is illustrated by our first data on the kidney and pancreas. For the kidney (15), a complex organ with many functional cell types, CD10^+^CD26^−^ podocytes, CD10^+^CD26^+^ parietal, CD29^+^CD34^+^CD105^+^ glomerular endothelial, CD29^−^CD34^+^CD105^+^ peritubular endothelial, Bowman’s capsular CD13^−^CD26^+^CD227^−^, CD10^+^CD13^−^CD26^+^CD227^+^ proximal tubular, and CD10^−^CD13^+^CD26^−^CD227^−^ distal tubular cells can be distinguished (**Figure 1B**). These various cell populations could then be isolated from resected tissue specimens by the use of any suitable CD antibodies or combinations of them. The CD26^+^ tubular cells could likely represent the normal counterpart of renal cell carcinoma, which is also stained for CD26 (**Figure 1B**). For the pancreas (16), insulin-producing β islet cells are positive for CD99R, for example, as inferred from serial sections stained separately by antibodies to insulin and CD99R (**Figure 1C**). The utility of CD maps argues for the eventual creation of an atlas compiling CD information for all organs of the human body.

### 1.2 Prostate principal components analysis plot

**Figure 2:**
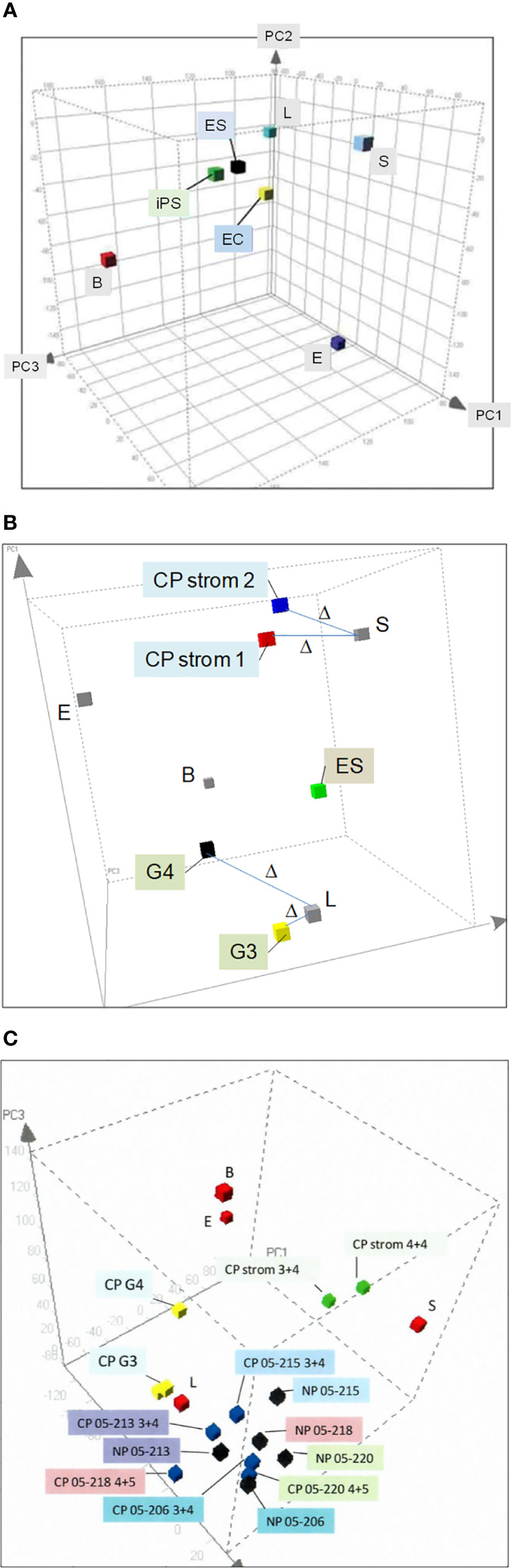
Prostate PCA plot. **(A)** The principal component axes of PC1, PC2, and PC3 are marked in the 3D display. The color cubes represent prostate cell-type transcriptome data points where L, luminal; S, stromal; B, basal; E, endothelial; ES, embryonic stem; EC, embryonal carcinoma, and iPS, induced pluripotent stem. **(B)** Transcriptome data points of CD26 G3 and G4 cancer and CD90 CP stromal are incorporated to show the gene expression difference from their respective normal counterparts, L and S. The distance between two data points, Δ, is a measure of differential gene expression. **(C)** The transcriptome data points of cells harvested by laser-capture microdissection, NP (black cubes) or CP (blue), are seen “intermingled” in one area of the PCA plot. In contrast, the transcriptome data points of L, B, and S are in separate areas. Unlike NP vs. CP, L vs. cancer G3, G4 (yellow) and S vs. CP stromal (green) are segregated in different areas.

We isolated prostate cell populations by flow cytometry or magnetic cell sorting (AutoMACS) (17) for analysis by DNA microarrays to obtain cell-type-specific transcriptomes (13). These transcriptome datasets were used to generate a prostate principal components analysis (PCA) space (18). In this 3D plot, the different cell types—luminal (L), stromal (S), basal (B), and endothelial (E)—as represented by their transcriptome data points are positioned on the periphery in relation to (cultured) stem cell types—embryonic stem (ES) (19), embryonal carcinoma (EC) (18), and induced pluripotent stem (iPS) (20), which are more toward the interior (**Figure 2A**). Transcriptome datasets of CD26 G3 and G4 cancer cells (8) and CD90 cancer-associated stromal cells (9) can be projected into this plot to visualize their extent of gene expression difference to their respective normal counterparts (**Figure 2B**). The separation between any two cell types is measured by Δ (18), where Δ = [(A_1_−B_1_)^2^+(A_2_−B_2_)^2^+(A_3_−B_3_)^2^]^1/2^, with the sub-indices being the coordinate values along the three principal components axes. Δ gives a measure of relatedness between any two cell types. Unrelated cell types with known unique functions are separated by large Δ, e.g., luminal vs. basal vs. stromal, whereas related ones are separated by smaller Δ, e.g., ES vs. EC vs. iPS, and luminal vs. G3 cancer. A bladder PCA space was likewise generated from the transcriptome data points of sorted bladder cell types (14). The bladder and prostate PCA plots showed that CD104 prostate basal and CD104 bladder basal cells were different in gene expression, i.e., separated by a large Δ, and are hence functionally different. They were also unlikely the candidate organ progenitor cell populations as indicated by the Δ between them and stem cells (14). In addition, no basal-like prostate cancer cell types were found (21). The PCA space is particularly powerful in showing transcriptome changes of cells as a result of gene transfection or cell–cell interaction (see below).

### 1.3 Cancer biomarkers

By transcriptomic comparison between G3 and G4 cancer and luminal cells, and between cancer-associated stromal and stromal cells, we identified suitable cancer biomarkers (22). Our obtained data demonstrate the advantage of cell sorting over laser-capture microdissection (23), a methodology frequently used in reports on differential gene expression between cancer (CP) and normal/benign (NP) (24, 25). **Figure 2C** shows the PCA plot of transcriptome data points of NP and CP cells microdissected from tissue specimens. There are no separate groupings of NP vs. CP. Examples of NP05-206, NP05-213, NP05-215, NP05-218, and NP05-220 are interspersed among the corresponding CP05-206 (GS3 + 4), CP05-213 (GS3 + 4), CP05-215 (GS3 + 4), CP05-218 (GS4 + 5), and CP05-220 (GS4 + 5), although the individual paired NP and CP are distinguished. There are no distinct groupings of the three 3 + 4 cases and the two 4 + 5 cases (cancer cells were captured from the G3 and G4 portions of the GS3 + 4 and GS4 + 5 tumor specimens, respectively). The Δ between NP05-213 and CP05-213 is even smaller than that between NP05-213 and NP05-218, the two closest placed NP. In contrast, placements of sorted cell data points show separation between normal and cancer, e.g., CD26 luminal vs. CD26 G3 cancer (CP G3) vs. CD26 G4 cancer (CP G4), with a smaller Δ between L and G3 than between L and G4. Additionally, CD90 cancer-associated stromal cells (CP strom 3 + 4 and CP strom 4 + 4) are distinguished from CD49a (NP) stromal cells. There was concordance between gene and protein expression of the CD antigens in the various sorted cell populations (26). A plausible explanation is that microdissected cells are more prone to be contaminated by untargeted cell types as detailed in our cell transcriptome analysis report (13). Such manual selection without any attempt at cell staining is less stringent than flow sorting. A new technology of single-cell RNA-sequencing is now being used to analyze cell types (27). Its known limitations include high variability/noise in the data obtained, low coverage (~10%) of the transcriptome from a single cell, and poor representation of lowly expressed transcripts.

### 1.4 Cancer differentiation-associated antigen AGR2

**Figure 3:**
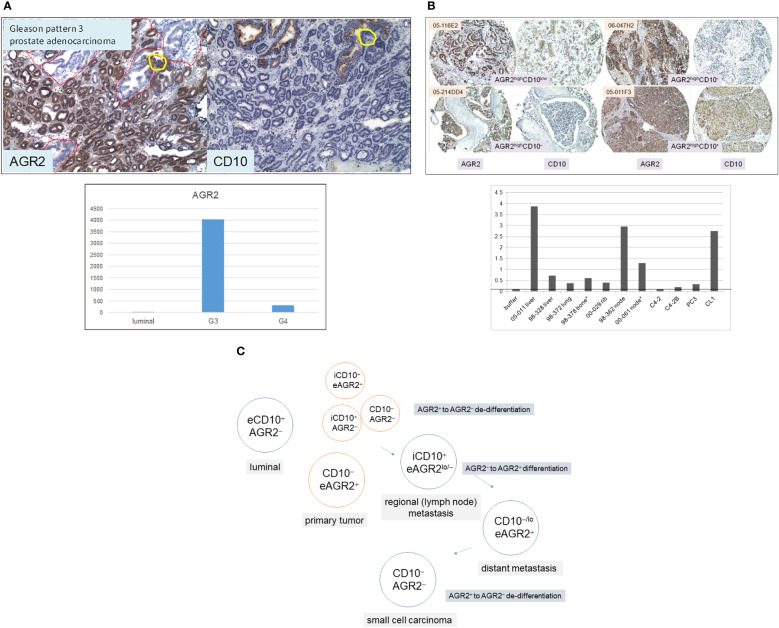
AGR2 and CD10 in prostate cancer. **(A)** Top: tumor glands in specimen 99-010D (mainly in lower 2/3 of the section) are AGR2^+^CD10^−^, whereas benign glands (some in the upper 1/3 of the section outlined in red) are AGR2^−^CD10^+^. The yellow circle outlines a portion of the benign gland with AGR2^+^CD10^−^ cells. Bottom: the histogram displays DNA microarray signal intensity values of AGR2 in luminal cells, G3, and G4 cancer cells. The values (*y*-axis) were retrieved from the Affymetrix microarray datasets archived in our SCGAP Urologic Epithelial Stem Cells Project (UESC) (28). They represent the average after clicking coalesce replicates and probe sets. Dataset query of this public database is described in Ref. 115. **(B)** Top: Shown are four representative sections of bone and soft tissue metastases identified by case numbers stained for AGR2 and CD10. Their AGR2/CD10 phenotypes are indicated. All four have strong AGR2 reactivity, while two have moderate to weak CD10 reactivity. Bottom: AGR2 was measured by ELISA in metastasis specimens (obtained from either surgery* or autopsy) and selected cell lines identified on the *x*-axis. The tumor specimens were minced and digested by collagenase, and the cell-free supernatants were analyzed as were media supernatant of cultured cell lines C4-2, C4-2B, PC3, and CL1. OD_405_ absorbance readings of the chromogenic dye are indicated on the *y*-axis. The line indicates the level obtained with buffer/media. **(C)** Prostate cells are tagged by AGR2 and CD10 expression for different AGR2/CD10 cancer phenotypes. The prefix “e” denotes the extracellular and “i” denotes the intracellular forms of these two proteins.

The Δ between G3 cancer and luminal is equivalent to ~200 differentially expressed genes with half upregulated and half downregulated in the cancer cells (8). Among the upregulated genes, the highest fold difference was one encoding anterior gradient 2 (AGR2, **Figure 3A**). It is a protein disulfide isomerase localized to the endoplasmic reticulum (ER) (29). AGR2 displays the following expression pattern: highest in G3 cancer cells, 10-fold lower in G4 cancer cells, high in prostatic intraepithelial neoplasia, and absent in luminal cells (8, 30). More notable, AGR2 is an adenocarcinoma antigen present in many types of solid tumor (29). As reported in pancreatic cancer, AGR2 was activated through ER stress that was induced experimentally by tunicamycin (31). ER stress constitutes an integral part of the cellular pro-inflammatory response. Mice with deleted Agr2 exhibited impairment in tumor formation (31). Inflammation and ER stress were also reported in prostate cancer development (32, 33). Among the downregulated genes was CD10 in agreement with the CD phenotyping result. CD10 displays a contrary expression pattern: absent in most G3 tumors, increase in higher Gleason tumors, and present in luminal cells (8, 11, 34). Prostate cancer cells can be phenotyped by AGR2 and CD10. Luminal cells display the phenotype of CD10^+^AGR2^−^ while cancer cells display that of CD10^−^AGR2^+^, and less frequently those of CD10^−^AGR2^−^, CD10^+^AGR2^+^, and CD10^+^AGR2^−^ (30, 35). Among the four cancer cell types, CD10^+^AGR2^lo/−^ is the predominant type found in local metastases (34–36). High-stage patients with this tumor phenotype at diagnosis have a ninefold lower recurrence-free survival than those with CD10^−^AGR2^+^ (35). At 60 months post-surgery, only 25% of these patients were recurrence-free compared to 85% of the CD10^−^AGR2^+^ cases. This suggests that CD10 functions in extracapsular escape of AGR2^lo^CD10^hi^ cancer cells. The cell line LNCaP and patient-derived xenograft (PDX) LuCaP 35 (37), established from node metastases, are both AGR2^−/lo^CD10^+^ (36). Therefore, AGR2/CD10 phenotyping of primary tumors can predict outcome, especially for patients with high-stage disease. In contrast to local metastasis, CD10^−/lo^AGR2^+^ is the predominant phenotype of distant metastases in bone and soft tissues (**Figure 3B**) (35). These metastases were shown to secrete AGR2 (**Figure 3B**). This suggests that AGR2 functions in the wider dissemination of cancer cells after exiting the prostate. Inhibition of AGR2 could abolish prostate cancer metastasis (38). Other genes are likely involved in AGR2-mediated metastatic spread since transfection of AGR2 into LNCaP cells produced multiple changes in the transcriptome (39). This supposition could be tested by comparing AGR2^−^ LNCaP with AGR2^+^ LNCaP for their invasive behavior. **Figure 3C** shows a schematic on gene expression changes involving AGR2 and CD10 in the disease course. The multiple AGR2/CD10 phenotypes indicate that their expression could change from AGR^+^ to AGR2^−^ and back to AGR2^+^, and from CD10^−^ to CD10^+^ and back to CD10^−^. In primary tumor, local metastasis, and distant metastasis, the differential functioning of CD10 and AGR2 is at play. Figuring out the difference between AGR2^+^CD10^−^ cancer cells in primary tumors and AGR2^+^CD10^−^ cancer cells in metastases is likely informative to solving the underlying mechanism. Cells of rare small cell carcinoma display the phenotype AGR2^−^CD10^−^ (19, 35). The difference between AGR2^−^CD10^−^ cells in primary tumor and AGR2^−^CD10^−^ cells of small cell carcinoma would also be informative. The association between cancer differentiation and AGR2 expression (high in G3) is also documented in breast cancer, with better patient outcome for tumors with elevated AGR2 expression (40, 41). Of note, both AGR2 and CD10 showcase abnormal protein trafficking in cancer. AGR2 is normally cytoplasmic but also secreted by, and present on, the cell surface of cancer cells (29, 42). CD10, normally a cell surface antigen, is also found in the cytoplasm of prostate cancer cells (34, 36), where it interacts with heat shock proteins (43). Two localization forms are thus possible for these proteins: intracellular (i) or extracellular (e), with eAGR2 and iCD10 being specific to cancer cells (as indicated in **Figure 3C**).

The biomarkers differentially expressed by cell types between cancer and normal were quantified in voided urine by multiplex RNA (44) and protein (45) platforms. A signal display of 18 RNA transcripts, due to their generally higher counts, could be used to detect high-grade cancer cases. The strong signal values for CD24, for example, were correlated to intense immunostaining of especially G5 tumors (44). Transcript signal from any shed iAGR2^+^ urothelial cells (see below) would impact its RNA test specificity. The best protein biomarker combination of epithelial AGR2, AGR3, CEAM5, stromal CD90, and SFRP4 produced an AUC value of 0.95 in distinguishing cancer from non-cancer (45). We developed protocols for amplification of urinary RNA and spin concentration of urinary protein for quantitative analysis by nanoString nCounter chips (46) and multiplex targeted mass spectrometry (45), respectively.

### 1.5 Prostate cancer cell types and stem cell transcription factor expression

From transcriptomes, we found two groupings of prostate cancer cells, one luminal-like around the luminal cell data point and the other less luminal-like/more stem-like around stem cell data points (21). The luminal grouping encompasses adenocarcinoma cell lines and cancer cells sorted from a G3 tumor (8), while the non-luminal grouping encompasses non-adenocarcinoma and small cell carcinoma cell lines, and cancer cells sorted from a G4 tumor (8). The cancer cell types are well represented by a family of PDX LuCaP lines (37). Small cell carcinoma LuCaP 145.1 in the stem-like grouping expresses stem cell transcription factors (scTFs) such as the core quartet of LIN28A, NANOG, POU5F1, and SOX2, as well as a low level (10-fold less) of β2-microglobulin (B2M) compared to the levels by non-stem cells (19, 39). In contrast, adenocarcinoma LuCaP 23.12 in the luminal-like grouping expresses only POU5F1, and a higher level of B2M (19). We demonstrated the link between scTF and low B2M by cloning the four scTFs from LuCaP 145.1 in expression vector pVITRO1*neo*, and transfecting them into human embryonic kidney fibroblasts (HEK293F). The resultant neo^R^ transfected cells showed a stem-like culture morphology (changing from that of fibroblasts) with a decrease in B2M expression (39). For comparison, the B2M level was not affected when the fibroblasts were transfected by the same vector containing immunoglobulin heavy- and light-chain genes (39). Other LuCaP lines express a subset of the four scTFs: LIN28A/POU5F1 in LuCaP 77 and LuCaP 73CR (19), LIN28A/NANOG^lo^/POU5F1/SOX2 in LuCaP 93 and LuCaP 173.2A (19), and SOX2 in LuCaP 49 (21). SOX2 is likely responsible for the neuroendocrine (NE) feature of small cell carcinoma. Transfection by SOX2 alone can convert fibroblasts into multipotent neuronal stem cells, which can then be induced to differentiate into several neuronal cell types (47). Many NE genes like enolase and chromogranin A are also expressed by stem cells as revealed by transcriptome dataset query (19). The increased expression of scTF from adenocarcinoma to non-adenocarcinoma and small cell carcinoma suggests a role in rendering cancer cells less differentiated toward more stem-like (48). The utility of scTF as biomarkers lies in the timely identification of relapsed patients who will most likely progress to small cell carcinoma. Today, nearly 20% of patients harbor small cell carcinoma after undergoing anti-androgen therapies (49). Operationally, stem-like cancer cells can be phenotyped as scTF^+^B2M^lo^ vs. scTF^−^B2M^hi^ of luminal-like cancer cells. Low B2M expression affects HLA-mediated interaction between cancer cells and immune cells (50).

### 1.6 Reprogramming of prostate cancer cells

**Figure 4:**
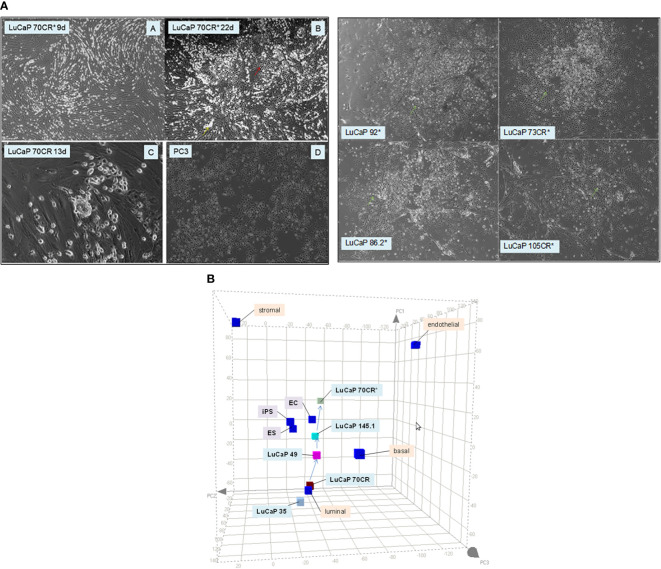
Reprogramming of adenocarcinoma cells. **(A)** The photomicrographs (left) show (A, B) scTF-transfected LuCaP 70CR* on culture days indicated, (C) mock-transfected LuCaP 70R, and (D) PC3. A similar cell appearance is seen between cultures of LuCaP 70CR* and PC3. The photomicrographs (right) show cultures of other similarly transfected LuCaP lines. **(B)** The PCA plot shows placements of the LuCaP 70CR and LuCaP 70CR* data points in relation to those of other cancer cell-type data points. A possible lineage (arrows) could be traced from luminal-like to more stem-like cancer cell types.

The grouping of cancer cells into luminal-like and stem-like suggested that cancer cells could undergo dedifferentiation, a recapitulation of luminal epithelial maturation in reverse. We demonstrated the lineage relationship of the two different cancer cell types by the use of reprogramming, an experimental process whereby iPS cells are obtained through scTF DNA transfection (51, 52). Freshly harvested LuCaP adenocarcinoma pieces were minced, digested by collagenase, and partitioned on Percoll gradient to remove mouse red blood cells. The resultant single cells were plated with irradiated mouse embryonic fibroblasts (MEFs, see below). LuCaP cells proliferated under this condition, and were passaged by adding trypsin. The culture-adapted tumor cells were resuspended for transfection by lentiviral vectors of the four scTFs (19). Five lines—LuCaP 70CR (CR = castration resistant variant), LuCaP 73CR, LuCaP 86.2, LuCaP 92, and LuCaP 105CR—were tested. In all cases, the resultant cells appeared dark compared to the untransfected or mock-transfected parental cells, and relatively smaller in size (**Figure 4A**). These proliferating cells were imaged at 1 month post-infection. **Figure 4B** shows the transcriptome data points of adenocarcinoma LuCaP 70CR and its reprogrammed derivative, LuCaP 70CR* (* to denote scTF-transfected). The Δ between LuCaP 70CR and LuCaP 70CR* was equivalent to that between CP stromal cells and their derived iPS-like cells obtained in a previous experiment (20). The LuCaP 70CR* data point was closest to that of LuCaP 145.1 in terms of Δ. Thus, adenocarcinoma prostate cancer cells can be reprogrammed to small cell carcinoma-like by scTF expression. In the PCA plot, a trace could be used to connect the luminal-like and stem-like cancer cell data points, outlining a prostate cancer differentiation pathway.

### 1.7 Stromal induction of stem cells

**Figure 5:**
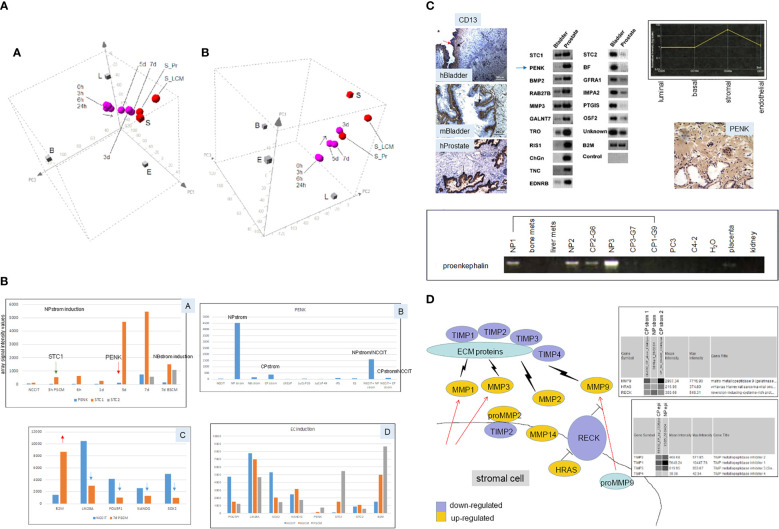
Stromal induction of EC cells. **(A)** The 3D PCA displays (in two orientations) show the “transcriptome migration” of NP stromal-induced NCCIT cells from that of stem cells (0 h) to that of stromal cells (S = CD49a-sorted, S_LCM = laser-capture microdissected, S_Pr = cultured) in a time course of 7 d. **(B)** Shown are selected genes induced in NCCT by stromal cell media. Panel A shows the temporal appearance of STC1, PENK, and STC2 by PSCM (prostate stromal media) vs. BSCM (bladder stromal media). Panel B shows the levels of PENK in different cell types. Panel C shows the downregulation of scTF (blue arrows) and upregulation of B2M (red arrow) in PSCM-induced NCCIT. Panel D shows a comparison between PSCM and BSCM induction. Array signal intensity values are indicated on the *y*-axis. **(C)** Top left: the three immunohistochemistry photomicrographs show human (h)Bladder and mouse (m)Bladder proximal lamina propria stained by CD13, while the human (h)Prostate stroma is negative, and the epithelial glands are positive (arrows). Top middle: the identified differentially expressed genes between CD49a prostate and CD13 bladder stromal cells are verified by RT-PCR. Arrowed is PENK. Top right: transcriptome dataset query shows PENK expression specific to prostate stromal cells, which was confirmed by immunostaining shown below. Bottom: RT-PCR results of tissue specimens show the absence of PENK in prostate cancer-associated stroma (CP1–CP3) compared to the normal counterpart (NP1–NP3). G6, G7, and G9 are Gleason sums. The lower amount in G6 could be due to residual NP tissue in the tumor specimen. PENK is absent in bone and liver metastases, PC3, C4-2 cancer cells, placenta, and kidney. **(D)** Schematic of the RECK pathway in stromal–epithelial interaction in prostate cancer. Decreased RECK expression leads to activation of MMPs and degradation of ECM proteins, allowing the release of tumor cells. Virtual Northern blot format shows array signals for MMP9, HRAS, and RECK in NP stromal vs. CP stromal (1 and 2 from two specimens), and for TIMPs in NP epithelial vs. CP epithelial.

Prostate stromal cells signal epithelial differentiation through secreted factors and cell contact (53). The process is an instructive induction in that the mesenchyme dictates the developmental fate of the epithelia, regardless of the source of progenitor cells, whether from the bladder, vagina, or urethra. Androgen influence is mediated through androgen receptor (AR)-positive mesenchyme (53). We showed this stromal induction by culture of scTF^+^B2M^lo^ EC cells, NCCIT (54), in conditioned media of prostate stromal cells (18). CD49a stromal cells were sorted from NP specimens, and cultured in fetal bovine serum (FBS)-supplemented media. Cell-free media supernatant (NPstrom) was added to NCCIT. Secreted factors in NPstrom induced NCCIT to differentiate into scTF^−^B2M^hi^ stromal-like cells indicated by colony morphology change and transcriptome analysis over 7 d (**Figure 5A**). Conditioned media of CD13 bladder stromal cells (**Figure 5A**, NBstrom) was also effective in inducing NCCIT (18), showing plasticity in stem cell response to different signaling. In both cases, downregulation of scTF and upregulation of B2M occurred in the resultant cells, concomitant with upregulation of either prostate or bladder stromal genes (**Figure 5B**). These stromal genes, particularly those encoding secreted protein molecules, were previously identified by a comparative transcriptome analysis between CD49a prostate stromal and CD13 bladder stromal cells (**Figure 5C**). The highest fold differentially expressed prostate gene was proenkephalin (PENK) (55). Dataset query showed that PENK expression was specific to prostate stromal cells, which was verified by immunostaining with a generated polyclonal antibody (**Figure 5C**). Prostate stromal PENK was not duly processed to enkephalin opioids, since antibodies to these peptide molecules were documented to not stain the stroma (56). PENK expression was found to be involved in development (57). The gene was induced in NCCIT/NPstrom at d 3 but not in NCCIT/NBstrom, confirming its prostate specificity (**Figure 5B**). Furthermore, PENK was not produced by CD90 prostate cancer-associated stromal cells (CPstrom) as determined from absent immunohistochemical staining and transcriptome dataset query (9). Accordingly, CPstrom did not induce PENK in NCCIT (**Figure 5B**). The absence of this abundant protein in CP stroma means that PENK signaling is missing in tumor. We postulate that abnormal cell–cell communication due to absent signaling could lead to faulty epithelial cell maturation. The difference between NPstrom and CPstrom induction of NCCIT appears to mimic the *in vivo* situation, for example, in the expression of matrix metalloproteinases (MMP) and tissue inhibitors of metalloproteinases (TIMP, **Figure 5D**) (18, 58). From comparing the CP-NCCIT and NP-NCCIT expression profiles, several genes associated with RECK (membrane-anchored reversion-inducing cysteine-rich protein with Kazal motifs) were differentially expressed. RECK downregulation has been detected in prostate and other cancers (59). Induction of MMP9, for example, was greater in CP-NCCIT. MMP9 was also higher in sorted CP vs. NP stromal cells as was HRAS (HRas GTPase), which promotes cell growth and division. MMPs are involved in the degradation of extracellular matrix (ECM) proteins, and associated with tumor cell dissemination. In contrast, RECK was more upregulated in NP-NCCIT than in CP-NCCIT, as was the MMP antagonist TIMP1. In responding NCCIT cells, NPstrom and NBstrom factors can modulate at least four scTFs simultaneously, and the EC cells can be induced to differentiate into stromal-like. We posit that scTF^+^B2M^lo^ stem-like prostate cancer cells could likewise be induced to undergo differentiation by stromal factors like PENK. The important aspect of heterotypic cell contact was more difficult to study experimentally due to the need to efficiently separate the two cell types post interaction for detail analysis other than immunostaining (18).

The CD cell typing and cell sorting allowed us to demonstrate other cell–cell interactions: (1) CD57 luminal cells cease synthesis of prostate-specific antigen (PSA) upon isolation; synthesis is restored by adding back CD49a stromal cells (60); (2) CD90 CP stromal cells appear to represent a less differentiated version of CD49a NP stromal cells as a result of stem cell factor influence from NCCIT cells in co-culture (61).

### 1.8 Effect of PENK on prostate cancer cells

**Figure 6:**
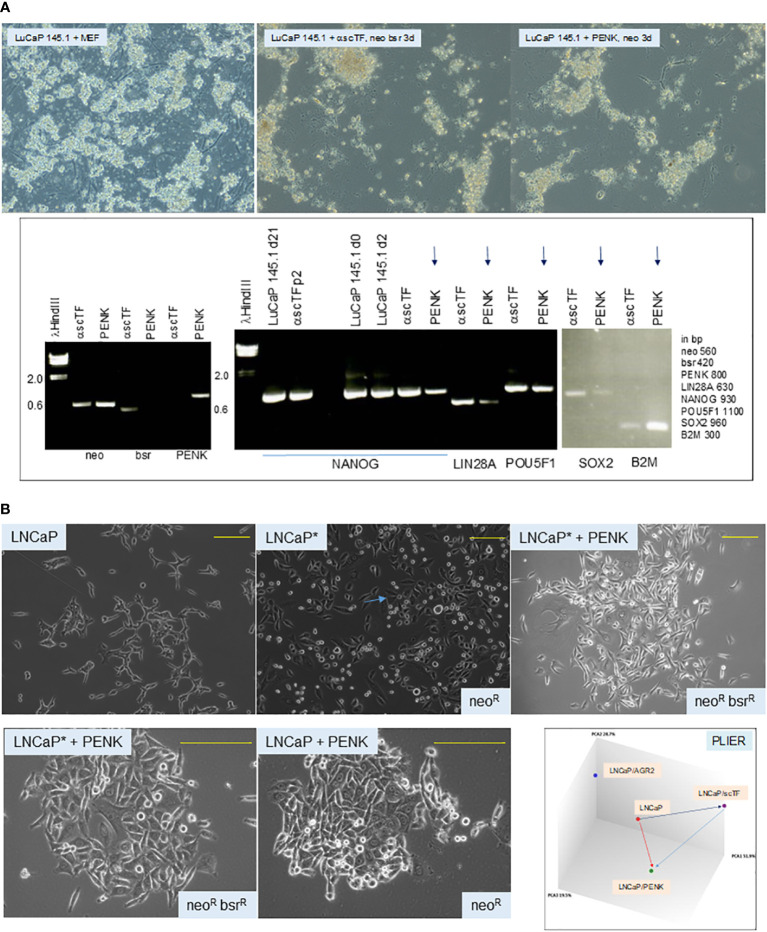
**(A)** Effect of PENK on LuCaP 145.1. The photomicrographs show LuCaP 145.1 cells with MEF and after transfection by PENK-*neo* or α-scTF-*bsr-neo* under appropriate drug selection. α-scTF vectors contained full-length antisense scTF genes, which showed no effect, and served as negative control. Drug-resistant cells proliferated for 3 d (with lysed MEF in the background) before harvest. The electropherogram confirms that PENK+ cells (LuCaP 145.1/PENK) were neo^+^bsr^−^PENK^+^ while PENK− cells (LuCaP 145.1/α-scTF) were neo^+^bsr^+^PENK^−^. The neo signal provides control for sample loading since it was expressed by both PENK+ and PENK− cells. B2M is typically used to serve as a house-keeping gene control, but in this case, it was differentially expressed between PENK+ and PENK− cells. PENK-transfected LuCaP 145.1 cells show downregulation of scTF and upregulation of B2M as gauged from the intensities of the reaction products in comparison to the corresponding ones seen in α-scTF-transfected LuCaP 145.1 (PENK−). The intensity difference for POU5F1 was not as large as this scTF is also expressed by non-stem-like LuCaP lines. The gel picture is a composite of two halves of a single run (bottom and top rows of gel loading wells with different background ethidium bromide staining). **(B)** Dedifferentiation and differentiation of cancer cells. The top photomicrographs show cultures of LNCaP, LNCaP*, and LNCaP*/PENK; the bottom photomicrographs show cultures of LNCaP*/PENK and LNCaP/PENK under a higher magnification (yellow bars). The diagram labeled PLIER shows relationships among the LNCaP, LNCaP/scTF = LNCaP*, and LNCaP/PENK data points. LNCaP/AGR2 shows the alteration in LNCaP transcriptome by AGR2 (Ref. 39).

To answer whether scTF^+^B2M^lo^ small cell carcinoma could respond to stromal factor signaling, we transfected PENK into LuCaP 145.1. The PENK vector was constructed by insertion of full-length PENK cDNA (from Kozak box sequence to stop codon) into pVITRO1*neo* (62). Freshly harvested LuCaP 145.1 tumor pieces were minced, digested with collagenase, and partitioned on Percoll gradient. The resultant single cells were plated on MEF. For transfection, the culture-adapted LuCaP 145.1 cells were resuspended for electroporation. The obtained neo^R^ cells were analyzed by reverse transcriptase-polymerase chain reaction (RT-PCR) at d3. A longer time point was not attempted because the absence of viable MEF (lysed by the added drug in selection) would be deleterious for the feeder-dependent cancer cells. Nevertheless, the PENK plasmid was subsequently found stably integrated into the host chromosome, so continuous drug selection was probably not necessary in long-term culture of LuCaP 145.1PENK^+^ clones with MEF. The neo^R^ LuCaP 145.1 cells showed downregulation of scTF and upregulation of B2M (**Figure 6A**), a response indicative of PENK being able to single-handedly alter the differentiation state of stem-like small cell carcinoma by targeting scTF.

### 1.9 Cancer cell differentiation and dedifferentiation

To show that cancer cells can undergo differentiation and dedifferentiation (62), we reprogrammed luminal-like scTF^−^B2M^hi^ LNCaP cells by scTF plasmid transfection. scTF transfection reduced the level of B2M, which was not seen on transfection of LNCaP by PENK or AGR2 (39). The resultant scTF^+^B2M^lo^/neo^R^/LNCaP* cells appeared small and darker than the parental LNCaP under light microscopy (**Figure 6B**). This cell appearance was similar to that observed in reprogrammed adenocarcinoma lines LuCaP 70CR*, LuCaP 73CR*, LuCaP 86.2*, LuCaP 92*, and LuCaP 105CR* (cf. **Figure 4A**). The neo^R^/LNCaP* cells were then transfected by plasmid vector pVITRO1*bsr*-PENK, and selected for resistance to blasticidin (bsr^R^). The neo^R^bsr^R^/LNCaP*/PENK^+^ cells appeared to have lost the “reprogrammed” cell morphology, and, instead, appeared like LNCaP transfected by PENK (**Figure 6B**). SOX2 was upregulated in LNCaP*, and then downregulated in LNCaP*/PENK^+^ (62). Note that PENK expression could also cause changes in the appearance of LNCaP cells: compare LNCaP with LNCaP/PENK^+^. LNCaP cancer cells, although being aneuploid and harboring a number of characterized mutations, are thus still capable of undergoing dedifferentiation by scTF, and differentiation by PENK with attendant changes in cell morphology and transcriptome (**Figure 6B**). Of relevance, we reported that PENK-positive NP stromal cells were not reprogrammed by scTF, i.e., refractory to reprogramming, while PENK-negative CP stromal cells were (20). These experiments show that PENK acts to antagonize scTF allowing NCCIT, LuCaP 145.1, and LNCaP* cells to exit the stem state. Our working hypothesis is that PENK could induce differentiation of any stem-like cancer cells such as the small cell carcinoma of many organs. In the disease course, cancer cells undergo gene expression changes with activation and inactivation of key transcription factors that control cell-state gene expression (63). We transfected PENK into HEK293F cells so that clinical grade quantities of the protein can be isolated from the culture media. It is also useful to develop PENK monoclonal antibodies. We can then test if immune-affinity purified PENK can directly modulate LuCaP 145.1 *in vitro*, and in xenografted animal hosts.

### 1.10 Effect of PENK on adenocarcinoma LuCaP 70CR

**Figure 7:**
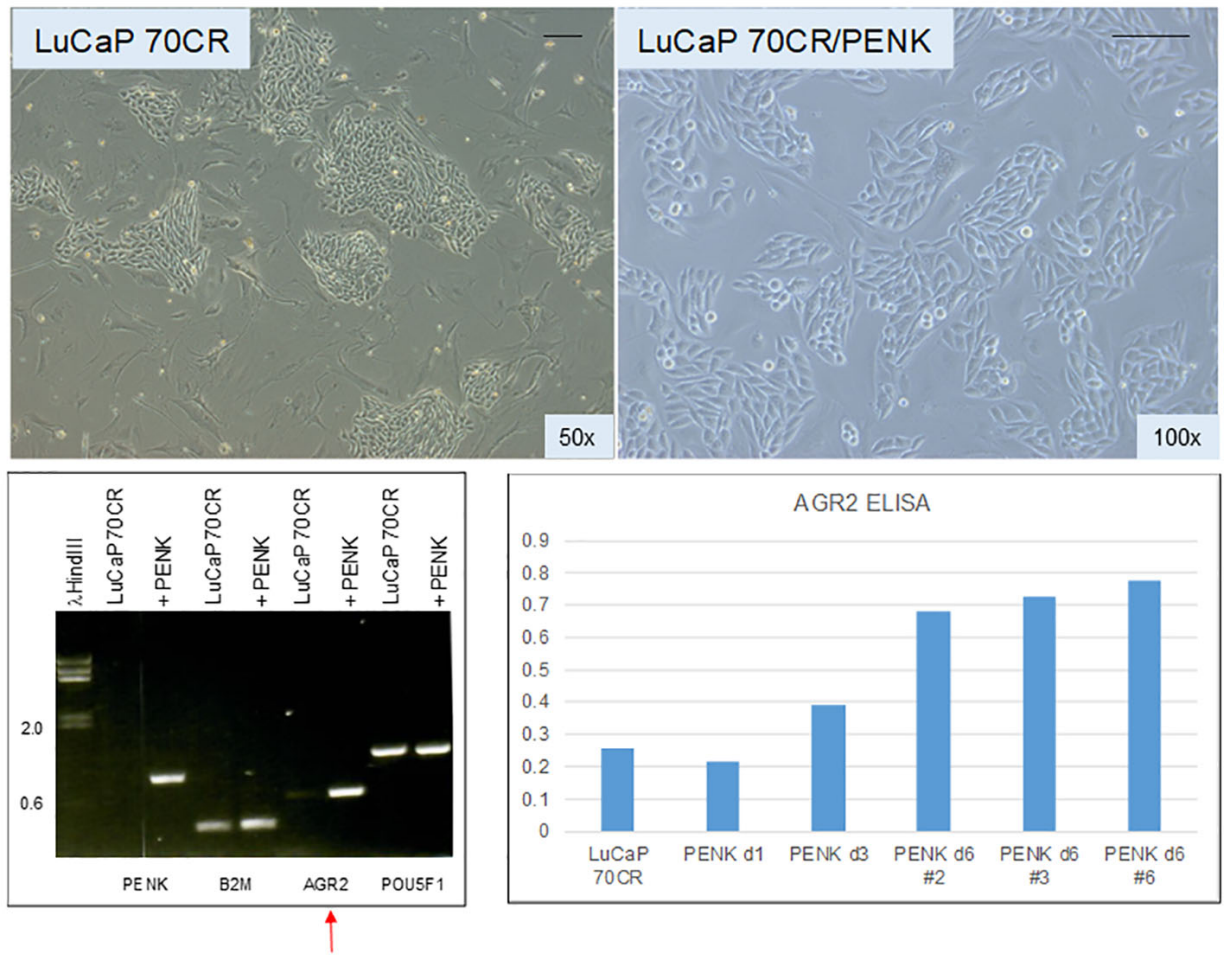
Effect of PENK on LuCaP 70CR. The photomicrographs show LuCaP 70CR before and after PENK transfection. The electropherogram shows an increase in the expression of AGR2 mRNA (arrow). Increased AGR2 expression was validated by measurement of secreted AGR2 in the culture media. The histogram is a representation of the optical density values (*y*-axis) from ELISA measurement. PENK d6 #2, 3, and 6 are three selected LuCaP 70CR/PENK cell clones analyzed from 1 to 6 d in culture.

Given the effect of PENK on LNCaP, PENK could likely have an effect on non-stem-like LuCaP cells. We transfected scTF^−^B2M^hi^ LuCaP 70CR by PENK (62). **Figure 7** shows LuCaP 70CR plated on MEF before and after PENK transfection. Unlike in LuCaP 145.1, PENK did not affect the expression of B2M and POU5F1 (the other three scTFs are not expressed by this luminal-like line) (19). Of note, PENK increased expression of AGR2 (**Figure 7**, electropherogram). The increase was confirmed by ELISA measurement of secreted AGR2 in the culture media of three cloned LuCaP 70CR/PENK cells (**Figure 7**, histogram). The elevated AGR2 expression was indicative of cancer cell differentiation induced by PENK. Being linked to differentiation, AGR2 expression is lowered through dedifferentiation as LuCaP 70CR was selected from LuCaP 70 by host castration.

### 1.11 Lineage model of prostate cancer cells

**Figure 8:**
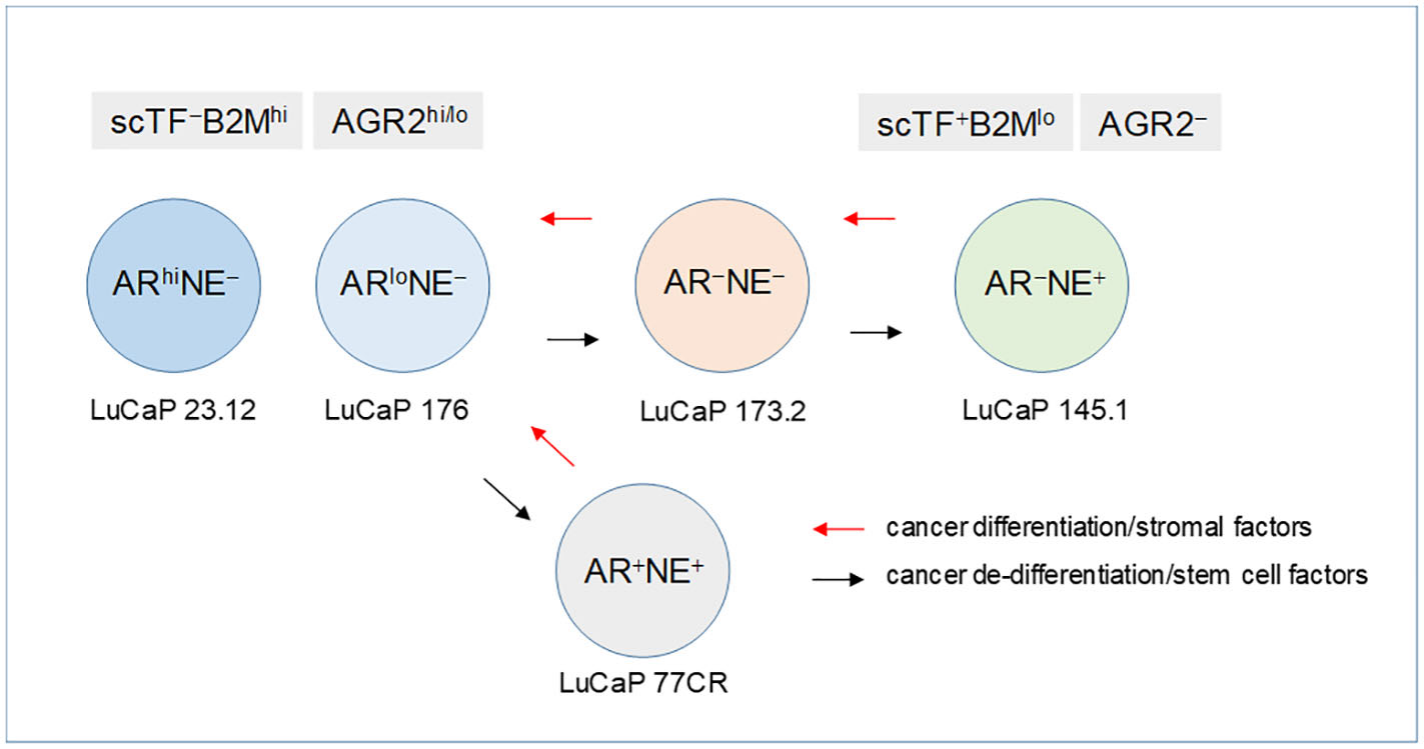
Lineage of prostate cancer cells. In this schematic, the different prostate cancer cell types are identified by AR and NE expression. The progression from AR^+^NE^−^ luminal-like to AR^−^NE^+^ stem-like is through the sequential activation of scTF, which is equivalent to reprogramming. Stem-like cancer cells respond to stromal factors such as PENK by undergoing differentiation changing from scTF^+^B2M^lo^ to scTF^−^B2M^hi^. The cell types are represented by different LuCaP lines. The AR^+^NE^+^ and AR^−^NE^−^ types represent intermediates that can become AR^−^NE^+^ from losing the AR program and gaining the NE program by the former, and gaining the NE program by the latter. The adenocarcinoma antigen AGR2 is associated with differentiation, from AGR2^hi/lo^ to AGR2^−^.

Using AR expression to denote luminal-like adenocarcinoma and NE expression to denote stem-like small cell carcinoma, prostate cancer differentiation (from NE^+^ stem-like to AR^+^ luminal-like) and dedifferentiation (from AR^+^ luminal-like to NE^+^ stem-like) can describe a lineage relationship among the cancer cell types (**Figure 8**). Luminal expression (AGR2^+^) is governed by AR signaling, while NE expression (AGR2^−^) in stem-like is due to SOX2. The LNCaP experiment supports the validity of this model of bi-directional changes: LNCaP → LNCaP* → LNCaP*/PENK ≅ LNCaP/PENK. In the LuCaP series (64), the AR^hi^NE^−^ type can be represented by LuCaP 23.12 (35, 70, and many others), AR^lo^NE^−^ by LuCaP 176 (and others), AR^−^NE^+^ by LuCaP 145.1 (93 and 145.2), AR^−^NE^−^ by LuCaP 173.2 (with squamous features, possibly activated by non-AR, non-NE signaling), and AR^+^NE^+^ by LuCaP 77CR. Note though that AGR2 expression appears linked to that of AR, and it is not absolute. AR-positive LuCaP 35 and LNCaP show low to null AGR2 expression (21). Variants derived from selection of LNCaP in androgen-depleted media, CL1, and CL2 show high AGR2 expression (65). How the AGR2 gene promoter is activated in the different cell types awaits to be answered.

## 2 Therapeutic treatments against differentiated and undifferentiated tumors

In early stages, prostate cancer can be managed with relatively high survival for patients (66). Patients diagnosed with organ-confined tumors can be treated by surgical resection, pinpoint radiation, or active surveillance if the tumor characteristics allow it. In later stages, prostate cancer can no longer be adequately managed, and effective treatment is limited. Patients with disseminated disease as indicated by rising PSA can be treated by targeting AR (67). However, many would fail, and the cancer becomes resistant to anti-androgen therapies. Therefore, new therapeutic targets are being sought. Our above discussion suggests that AGR2 and scTF could be viable targets.

### 2.1 High expression of AGR2 in prostate cancer metastases

We carried out AGR2 immunostaining on tissue microarrays (TMAs): UWTMA22 metastases of donor autopsies containing 248 cores were sampled from 124 sites in 23 patients treated by androgen ablation and other systemic therapies; UWTMA46 of 24 LuCaP lines were established from these and other metastases (37). Tumor cells in bone and soft tissue metastases were scored AGR2^hi^ by stain intensity (cf. **Figure 3A**). The staining was uniform, and in agreement between tumors and their LuCaP lines (35). Only NE small cell carcinoma samples were unstained. The AGR2 levels in metastases and LuCaP lines were corroborated by DNA microarray analysis (35). Given these results, targeting AGR2 would have an impact in treating metastatic diseases since 96.4% of lesions are AGR2^+^ adenocarcinoma against 0.7% AGR2^−^ small cell carcinoma and 2.9% AGR2^+^/AGR2^−^ mixed carcinoma (68).

### 2.2 Prevalence of AGR2 expression in solid tumors

We immunostained bladder cancer TMA of primary tumors and corresponding lymph node metastases sampled from 152 lymph node-positive cases treated by cystectomy and pelvic lymphadenectomy (42). The bladder urothelium was uniformly stained for AGR2 at moderate intensity compared to the intense staining of prostate tumors. This difference was corroborated by DNA microarray data where the AGR2 expression level was 40× lower in CD9 urothelial cells than CD26 prostate cancer cells (13). Approximately 25% of the tumors showed AGR2 immunostaining. In many cases, the cancer staining appeared stronger than that of uninvolved urothelium. For a majority of bladder cancer, malignant transformation led to AGR2 loss. For lymph node metastases, 44% showed AGR2 staining. There were cases in which AGR2 staining was not detected in the primary tumors but was in the lymph nodes (42). No correlation was found between patient survival and AGR2 expression in this cohort (42). These bladder tumors were previously stained for CD10, and CD10 was found to correlate with good outcomes (69), in contrast to prostate cancer. An example of the AGR2^−^CD10^+^ phenotype was identified by microarray data of sorted CD9 cancer cells (13). The difference between these bladder cancer cells and AGR2^−^CD10^+^ prostate cancer cells could be informative on the molecular mechanism behind their differing influence on patient survival.

We stained 1,202 non-small cell lung cancer (NSCLC) sampled from a cohort of 400 patients (70). The tumor types tested included adenocarcinoma, squamous carcinoma, and large cell carcinoma, plus some NE and adenosquamous carcinomas. The lung epithelium showed uniform staining. Adenocarcinomas showed slightly stronger staining on average than squamous or large cell carcinomas. Only a very small percentage showed no staining. AGR2 expression was inversely correlated with grade, similar to prostate cancer. No differences were seen in the staining of primary sites, lymph node, and distant metastases. When segregated by tumor types, high AGR2 expression was more pronounced in adenocarcinoma, also indicative of AGR2 with cancer differentiation. AGR2 was a significant predictor for patients under 65 in that higher levels were associated with poorer survival (70). Previously, CD10 was reported in ~20% of NSCLC but with no diagnostic value (71).

The purpose of the above data presentation is to highlight the prevalence of AGR2 in cancer. In prostate, expression is absent in normal but high in cancer. In bladder, expression is present in normal and absent in 75% of cancer. In lung, expression is present in both normal and cancer. AGR2 is correlated with better survival in prostate cancer, poorer survival in lung cancer, and neither in bladder cancer. CD10 is correlated with poor survival in prostate cancer, better survival in bladder cancer, and neither in lung cancer. Finding a basis for these disparate survival correlations may hinge on the interaction of AGR2 (and CD10) with other molecules in cancer cells. Comparative analysis between AGR2^−^ luminal/AGR2^+^ prostate cancer, AGR2^+^ urothelial/AGR2^−^ bladder cancer, AGR2^+^ urothelial/AGR2^+^ bladder cancer, and AGR2^+^ bronchial/AGR2^+^ lung cancer may lead to identifying these interactions.

### 2.3 Cancer specificity of eAGR2

Faint staining of the stroma next to prostate tumor glands (cf. **Figure 3A**) could indicate that AGR2 was secreted by AGR2^+^ cancer cells (35). The absence of staining in the lamina propria below the urothelium could indicate that no AGR2 was secreted by AGR2^+^ urothelial cells (42). No appreciable amount of AGR2 was measured in voided urine samples from young healthy female donors collected on separate days (42). The positive control was tissue digestion media of LuCaP 23.12, which had a 25-fold higher level of secreted AGR2 than buffer. That little AGR2 released into urine was supported by query of urine proteome databases. AGR2 was not found in the *UrinePA-PeptideAtlas* archive of 2,500 proteins (72), nor the core urinary proteome of 587 proteins scored from healthy people (73). On the other hand, AGR2 was secreted by AGR2^+^ bladder cancer cells. Urine from a bladder cancer patient scored 7.5-fold higher than control. Five of 20 patients in a study cohort scored positive for urinary AGR2, which matched the percentage of bladder cancer being positive for AGR2 (42). *PeptideAtlas* query also yielded very low AGR2 peptide counts from the blood of healthy people given that the bronchial epithelium expresses AGR2. When measured by targeted mass spectrometry proteomics, the serum level of AGR2 was near background (74). On the other hand, sera of five prostate cancer patients were tested positive for AGR2 with a good correlation between the amounts of AGR2 (in pg/mL) and PSA (in ng/mL), a result not possible if there was a base level of AGR2. Like bladder cancer cells, lung cancer cells were measured to secrete high levels of AGR2 in culture (75). These results show that secretion of AGR2 and, by extension, eAGR2 expression is limited to cancer.

### 2.4 Tumor localization of anti-AGR2

**Figure 9:**
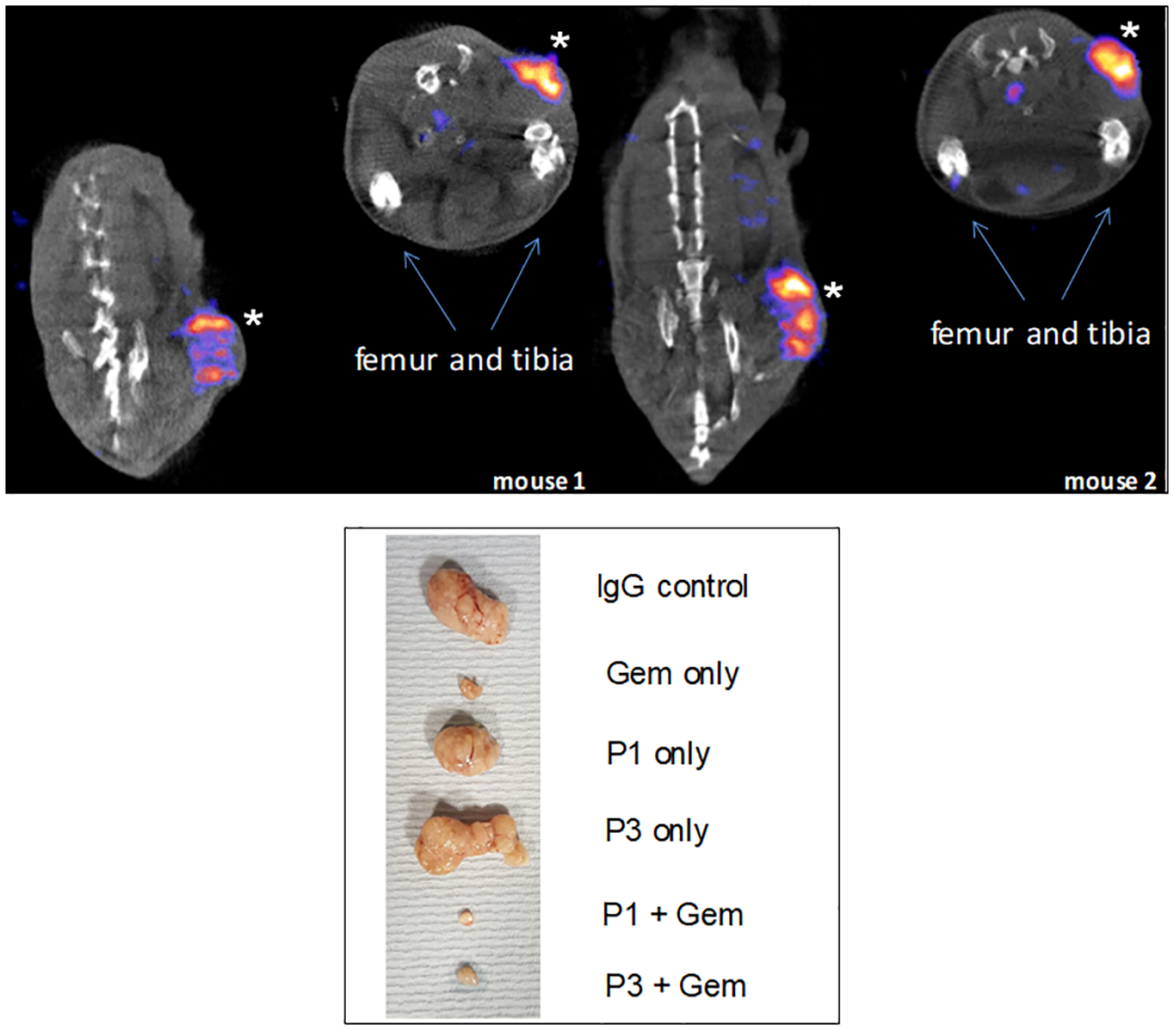
Specific tumor targeting. Top: mice were implanted with Agr2-positive DT6606 mouse pancreatic cancer cells. At post-injection of radiolabeled ^111^In-anti-AGR2, the tumors were strongly labeled (marked by *). No significant labeling could be detected in iAgr2-positive normal tissues. Bottom: shown are implanted pancreatic cancer PDX sizes in response to treatment with anti-AGR2 P1 (P1G4), P3 (P3A5), alone or in combination with Gemcitabine (Gem). The antibodies alone produced no effect; the best tumor growth suppression was achieved in the P1 + Gem group.

We generated mouse monoclonal antibodies, P1G4 (mIgG1) and P3A5 (mIgG2a), to AGR2 (65). Radiolabeled P3A5 was injected into mice bearing implanted murine Agr2^+^ DT6606 pancreatic cancer cells (76). At post-injection, strong labeling of the eAgr2^+^ tumors was detected but not iAgr2^+^ bladder or lung (77). The radio-imaging data confirmed cancer cell surface expression and cancer specificity of eAgr2 (**Figure 9**). P3A5 recognizes both human AGR2 and mouse Agr2, which means that a similar result could be obtained in human patients, i.e., localization to eAGR2^+^ pancreatic tumors but not iAGR2^+^ lung or bladder. There is a strong likelihood that anti-AGR2 would have minimal effect on normal cells that do not express eAGR2. The generation of AGR2 monoclonals supports this argument. In the immunized mouse hosts, the resulting circulating anti-AGR2 IgG would have attacked any Agr2-expressing cells to cause systemic harm or death. Instead, at sacrifice to harvest the splenic cells, there were no visible damages in any of the internal organs examined (65).

### 2.5 Enhancement in drug-induced tumor inhibition by anti-AGR2

We reported that a combination of the chemo-drug Gemcitabine (Gem) and P1G4 was found to result in decreased growth of implanted eAGR2^+^ human pancreatic tumor xenografts compared to Gem alone (**Figure 9**) (77). On cessation of drug administration, the tumors started to increase in size and proliferated at a faster rate than those treated by P1G4 + Gem. This growth difference was confirmed by the immunostaining of proliferation marker Ki67 (77). The effect was epitope-specific as P3A5 produced no such enhanced tumor inhibition. The serum levels of tumor secreted AGR2 correlated with tumor burden. Internal organs examined at study end also showed no damages.

### 2.6 Therapeutic targeting of cell surface AGR2

Being cancer-specific makes eAGR2 a unique tumor-associated antigen (TAA) that furnishes a singular target for immunotherapeutics. The successful cancer treatment by antibodies to HER2/EGFR (CD340), which is amplified in a subset of breast cancer, validates this strategy (79). Targeting eAGR2 on cancer cells would spare iAGR2^+^ normal cells. Antibodies trigger cell lysis by antibody-dependent cellular cytotoxicity (ADCC) (80) and complement-dependent cytotoxicity (CDC) (81). These processes involve interaction of immune system T cells and serum complements with cancer cell-bound IgG molecules. We replaced the mouse constant domains of AGR2 antibodies by the corresponding human C_γ_ and C_κ_ domains via recombinant DNA (77). The mouse variable V_H_ and V_κ_ of P3A5 and P1G4 sequences were joined respectively to the human constant C_γ_ and C_κ_ cloned from donated white blood cells. HEK293F cells were transfected with plasmid vector of the human:mouse chimeric H and L chain genes. G418 drug-resistant (neo^R^) transfected cells produced equivalent amounts of H and L mRNA, and equivalent amounts of H and L proteins (77). In all, we obtained chimeric hIgG1, hIgG2, and hIgG4 for both P1G4 and P3A5. C_γ_3 cDNA was not found in the blood sample used; if needed, it can be cloned from commercially available IgG3-producing cell lines. Since different IgGs may interact with multiple immune system components (82), employing all IgG isotypes in treatment may prove advantageous.

AGR2 binding was assayed for chimeric antibodies in the cell-free media. In the assay, P1G4 was used to capture AGR2 secreted from LuCaP cells followed by P3A5 (positive control) or the chimeric IgG, then HRP-conjugated anti-mouse IgG2a or anti-human IgG for detection. The chimeric IgG1 (and IgG2 and IgG4) and P3A5 detected equally well the different amounts of AGR2 from LuCaP 35CR, LuCaP 86.2, LuCaP 105, and LuCaP 147 (77). Media from serially passaged cultures showed that IgG synthesis continued from the stably integrated transgenes. The IgG-producing clones were weaned from serum supplement, and cultured in the absence of toxic G418. The serum-free culture media contained few other proteins (293F being non-secretory compared to hybridoma cells), and the secreted 150-kDa IgG proteins were concentrated by simple spin filtration.

In our earlier work, target cancer cells were exposed to chimeric antibodies with human serum or peripheral blood leukocytes (83, 84). In CDC, the chimeric antibodies produced a higher cytotoxicity at all complement dilutions. In ADCC, the chimeric antibodies produced a greater degree of cytolysis at a concentration 100× lower than the mouse antibodies. ADCC was observed down to a 3:1 ratio of leukocytes to cancer cells. Cell killing was not observed against cells lacking the targeted TAA. PC3 cells with a low cell surface expression of eAGR2 were incubated with freshly donated human serum and antibodies. The effect on cell growth was not seen with human serum only or with mouse P3A5. Cell growth was inhibited by chimeric IgG1, IgG2, and IgG4 plus serum, resulting in culture well surface showing large areas devoid of cells, and a floating mass of cell debris after 3 d (77).

In prostate cancer immunotherapeutics, antigens such as PSA, PAP, PSCA, MUC1, and PAGE/GAGE have been used to stimulate T cell-mediated immunity against prostate cancer (85). A lack of consistent results could in part be attributed to their expression not being restricted to the prostate. For example, PSCA (prostate stem cell antigen) was found expressed also by the bladder, colon, kidney, and stomach (86). The trials of PSA (PROSTVAC-VF) and PAP (prostatic acid phosphatase, Provenge) were inconclusive with some small increase in survival (85). Strategies to interfere immune checkpoint factors (CTLA-4, PD-1, and PD-L1) with the intention of boosting anti-tumor T cell response were not particularly successful. One expected side effect was immune-related adverse events due to tissue damage caused by hyper-activated T cells (87). These therapies were beset by response monitoring, although a subset of patients with advanced disease seemed to respond. Immunotherapy with TAA antibodies, in contrast, would not require tinkering with the immune system to achieve a clinical benefit.

An antibody–drug conjugate (ADC) to prostate-specific membrane antigen (PSMA) TAA was reported to produce clinically relevant decline in serum PSA and circulating tumor cell counts in metastatic castration-resistant, taxane-experienced, and chemo-naive patients (88). Adverse effects in some patients were neutropenia, fatigue, electrolyte imbalance, anemia, and neuropathy. A small number died from progression. PSMA encodes a membrane metalloenzyme found in many organs (89). More important, not all prostate cancer cells express PSMA. This ADC was shown to be less (or not) effective against tumors with low or null PSMA expression (90). The downside is that its application would lead to selection of PSMA-negative cancer. In contrast, our data showed that most prostate cancer metastases were positive for AGR2. Anti-AGR2, unlike anti-PSMA, would be effective for patients with prostate and, say, lung metastases where both contain eAGR2^+^ cancer cells. The rationale of our developing anti-AGR2 is based on eAGR2 being tumor-specific and metastatic prostate cancer cells expressing high levels of it. The availability of two AGR2 antibodies could overcome potential allelic differences in either epitope. Anti-AGR2 and anti-PSMA when used in combination would be effective against PSMA^−^AGR2^+^, PSMA^+^AGR2^−^, and PSMA^+^AGR2^+^ tumors.

Direct antigenic stimulation of T cells in CAR-T cell therapy provides another option (91). The V domains of P1G4 and P3A5 are joined to T-cell activator molecules to allow direct homing of T cells to eAGR2^+^ tumor cells. In the future, one could induce patient-derived iPS cells to differentiate into functional dendritic cells using bone marrow environment and marrow stromal cells. The *in vitro*-derived dendritic cells can then be primed by AGR2 for maturation. In a published report, AGR2 was transfected into dendritic cells or used to stimulate them to generate T cells capable of lysing AGR2^+^ (colorectal) cancer cells (92).

Last, eAGR2 expression allows for the development of a cancer vaccine. After first-line treatment, patients can be immunized by AGR2 (say, via an RNA immunogen). Any emerging cancer cells with eAGR2 subsequently are eliminated. Antibodies secreted by the resultant immune cells could inhibit the functioning of secreted AGR2 in cancer spread, and neutralize the deleterious effect of AGR2 in inducing programmed cell death of susceptible cells (93). Normally secreted AGR2 probably acts in early development to signal stem cell differentiation such as found in the process of limb regeneration of lower vertebrates (94). Thus, cancer patients immunized against AGR2 would be fully protected from recurrence and metastasis. The cancer specificity of AGR2 also means that early detection through imaging is possible since only eAGR2^+^ cancer cells would take up injected labeled antibodies as shown in our mouse study.

### 2.7 Differentiation therapy against stem-like tumors

The goal of differentiation therapy is to remove the block in terminal differentiation of cancer cells by supplying the missing signaling (95). It is foundationally based on searching for pharmaceutical chemicals that can allow cancer cells to undergo differentiation. Retinoic acid (RA) was found highly effective in treating acute promyelocytic leukemia (PML) (96). RA disarms the disease-specific oncoprotein PML-RARα that prevents myelocytic maturation. For other tumors, the challenge is to find a similarly effective drug for each. Other than PML, the specific cancer differentiation blocks are generally unknown. The question remains if this treatment strategy could be applied to solid tumors (97). Likelihood of success can be gauged from the drug treatment of testicular cancer. Germ cell tumor is a frequent solid cancer in young men. It can be cured by chemotherapy but with significant toxicity, while chemoresistance often leads to relapse and metastasis. EC is a major component of these tumors and could represent the hypothetical cancer stem cell. From a drug screen, thioridazine was found to have an inhibitory effect on cancer cells (98). On exposure to this compound, EC cells no longer formed ES-like colonies in culture, and appeared fibroblastic. POU5F1 was downregulated (99). The treated cells exhibited reduced tumorigenic potential, lowered proliferation, and decreased anchorage independence. Extended survival after drug intervention was seen in mouse models (99). Gene expression of the treated EC cells was indicative of differentiation. As described above, NPstrom and NBstrom produced a comparable effect on EC cells with change in culture morphology and downregulation of POU5F1 and other scTFs. Decreased tumorigenicity by thioridazine in stem-like PC3 cells was reported to involve AMPK inhibition (100). To date, compounds like thioridazine have been explored to treat multiple types of cancer with stem features (101, 102). Stem cell signature has been documented for some time in solid tumors (103). For clinical application, natural products like PENK would have an advantage over thioridazine, which is a potent antipsychotic with undesirable side effects.

Loss of cancer differentiation from luminal-like to stem-like leads to a less treatable disease as targetable markers such as differentiation-associated AGR2 and AR are no longer available. In their place, scTFs are the logical candidates for targeting since they are the drivers of lethal cancer. Transcription factors, being localized in the nucleus, are deemed undruggable. However, we have shown the effect of protein factors from stromal cells on scTF expression in stem-like cancer cells: EC, small cell carcinoma LuCaP 145.1, and reprogrammed LNCaP*. Prostate cancer would remain manageable if the transition from luminal-like to stem-like can be prevented or reversed.

### 2.8 Could normal cells be derived from differentiation of cancer cells?

**Figure 10:**
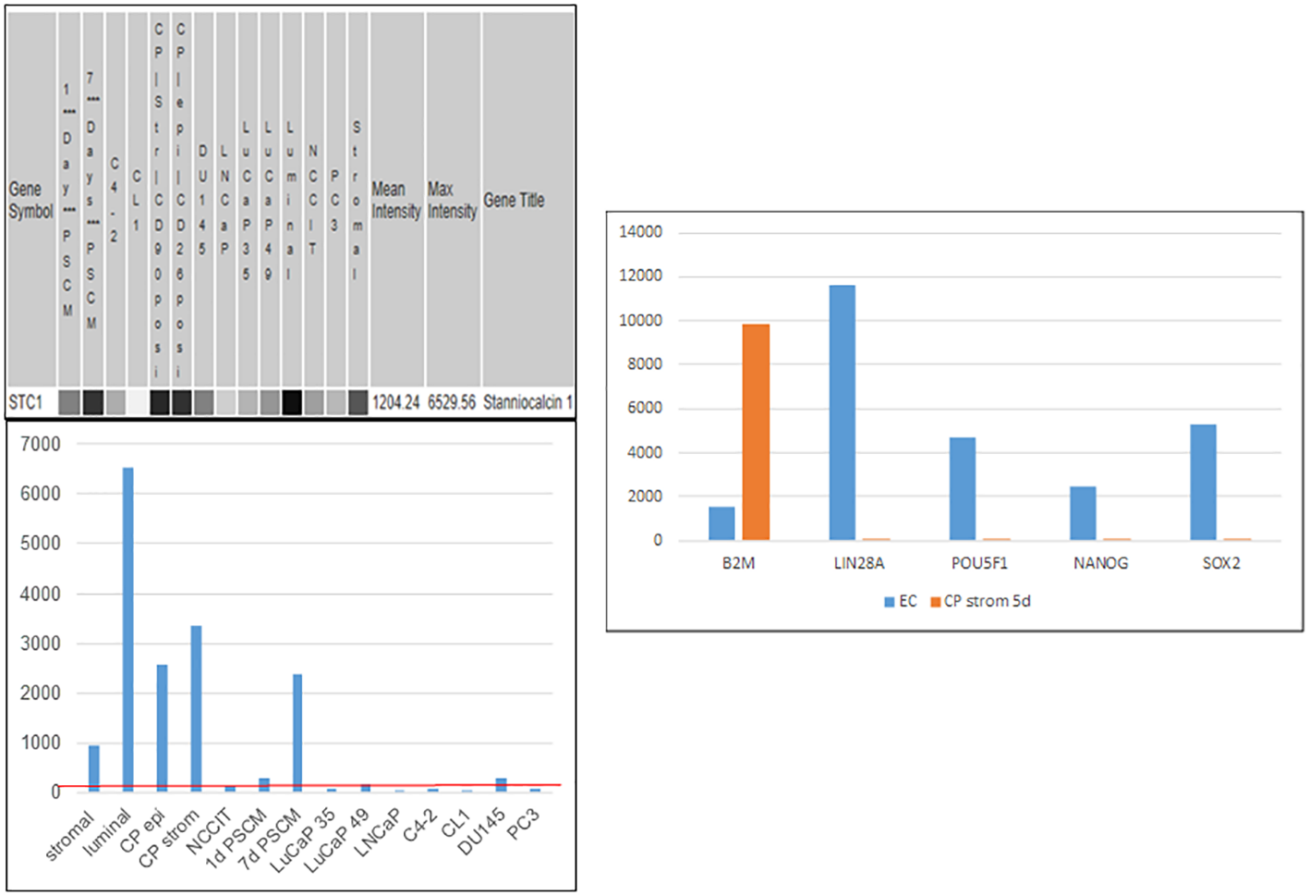
Stanniocalcin 1. Left: array signal intensity values were retrieved from transcriptome datasets (top) in UESC and displayed in histogram format (bottom). The red line shows low expression of STC1 in the cancer cell lines and xenografts listed, as well as NCCIT. Right: downregulation of scTF and upregulation of B2M were seen with induction by CPstrom at d5. Unlike NP stromal cells, CP stromal cells lack expression of PENK but not STC1 (histogram entry #4).

Like PENK, stanniocalcins STC1 and STC2 are genes involved in organ development (104, 105) encoding secreted proteins differentially expressed between prostate and bladder (55). STC1 was induced in NCCIT by NPstrom at <1 d, preceding PENK. STC1 was upregulated more than STC2 by NPstrom while STC2 was upregulated more than STC1 by NBstrom in correspondence to their relative levels in these stromal cells (18) (cf. **Figure 5C**). Thus, EC cells respond to both prostate and bladder factors to produce progeny with distinct gene expression: PENK^+^/STC1^hi^/STC2^lo^ vs. PENK^−^/STC1^lo^/STC2^hi^, respectively. Unlike PENK, STC1 was also expressed by epithelial cells. It showed a trend toward decreased expression from luminal to G3 cancer, to cancer cell lines and LuCaP lines; and from NP stromal to CP stromal (62) (**Figure 10**). Downregulation of STC1 could be a biomarker of advanced diseases. In signaling, STC1 and PENK could cooperate to induce stem/progenitor cells. STC1^+^ CPstrom (isolated from a GS3 + 3 tumor) lacking PENK could still change NCCIT from scTF^+^B2M^lo^ to scTF^−^B2M^hi^ (58). STC1 in CPstrom could thus be responsible for the NCCIT response and the differentiated state of G3 tumors. Less differentiated G4 and G5 tumors could be due to lower levels of STC proteins in their associated stromal cells. PENK, therefore, is not the only factor in differentiation but with contribution from others such as the STC proteins. We can test by transfection of LuCaP 145.1 with STC1 first and then PENK to mimic the induced expression sequence in NCCIT/NPstrom, followed by PCA plotting of the LuCaP 145.1/STC^+^, LuCaP 145.1/STC1^+^PENK^+^ vs. LuCaP 145.1/PENK^+^ transcriptome data points. A plausible outcome is that stem-like cancer cells could be induced into normal-like cells as seen in NCCIT + NPstrom. We can also monitor LuCaP 145.1 cells cultured in NPstrom or NBstrom for 7 d as in the study with NCCIT cells.

Our proposed therapeutic strategy is to restore the differentiation process by refurbishing the signaling molecules absent in prostate cancer. Future research will answer the following. Do all poorly differentiated tumors possess the stem-like scTF^+^B2M^lo^ phenotype? Can all stem-like cancer cells respond to stromal signaling? Can PENK promote differentiation of scTF^+^B2M^lo^ lung small cell cancer or bladder small cell carcinoma in addition to germ cell tumor and prostate small cell carcinoma? To tackle these issues, the following research tools are of use.

### 2.9 Adaptation of xenograft cells to *in vitro* culture

To date, many more representative human prostate cancer cells are available as PDX. It would be preferable to propagate them in culture. The LuCaP lines were established by UW Urology from tumor samples procured from surgery and donor autopsy (106). Tumor materials were implanted in severe combined immunodeficient male mice. Tumor take was based on serial passages *in vivo*. More than 50 lines have been analyzed with regard to gene expression and mutations (37, 64). The main drawback in their being used for experimentation lies in their time-consuming laborious preparation (107). While a small number of prostate cancer PDX have been successfully grown in culture as cell lines (108), this approach is haphazard. Instead, we developed a more reliable one where any PDX line can be propagated *in vitro* as well as frozen for long-term storage (19, 62). The *in vitro*-adapted LuCaP cells can be passaged by trypsin, and resuspended for manipulations such as gene transfection (39, 62). LuCaP cells grown and passaged as spheroids *in vitro* (109–111) would still be required to be dissociated to allow more individual cells to be transfected (given the efficiency of transfection of 10^−4^), and to interact and contact with other cell types in co-culture.

LuCaP 23 was one of the first reported lines with sister lines derived from lymph node metastases (LuCaP 23.1 and LuCaP 23.8) and a liver metastasis (LuCaP 23.12) of a single donor (106). The implanted tumors showed a glandular histology positive for PSA, and had a doubling time of 11 to 21 d. The PDX cells responded to androgen deprivation when passaged in castrated mice with decreases in PSA synthesis and tumor size. In time, the tumor cells became androgen independent. This is a convenient way to obtain CR variants—LuCaP 23.1CR. The glandular appearance and PSA synthesis are characteristic of adenocarcinoma. Approximately 90% of the obtained LuCaP lines are adenocarcinoma (37). The remainder include PSA-negative, non-glandular small cell carcinoma with NE features such as LuCaP 49 (112) and LuCaP 145.1. These cancer cells are insensitive to androgen deprivation, express no PSA and AR, and show rapid growth with a comparatively shorter doubling time of 6.5 d. Many LuCaP lines have a complex karyotype and loss of heterozygosity in certain chromosomes (64, 78). Some exhibit a hypermutator phenotype (78). Their gene expression, however, remains unchanged throughout multiple passages (in mice), for example, as shown for adenocarcinoma LuCaP 35 at p64, p71, p79, p94, and p99, and LuCaP 49 at p40, p45, p47, p49, and p59 (21).

Freshly harvested excess xenografts weighing 100–500 mg are minced and digested by collagenase (type 1, ThermoFisher) in 3 mL of 5% FBS-supplemented RPMI1640 media with ROCK inhibitor (compound Y-27632, StemCell) for several hours at room temperature on a low-speed magnetic stirrer. The digestion media is diluted by an equal volume of Hanks’ balanced salt solution (HBSS), filtered through a cell strainer. The cells are resuspended in HBSS. The cell-free supernatant is saved, which can be used to measure cancer-secreted AGR2 (65, 93), or used in co-culture to determine the effects of cancer-secreted proteins on other cells such as AGR2 in the induction of apoptosis of normal stromal cells (93). A Percoll discontinuous density gradient is used to remove mouse cells in the tumor samples. Any residual mouse fibroblasts would overtake the culture. Cancer epithelial cells are separated from mouse fibroblasts by their higher specific gravity (ρ = 1.070) (60). Small cell carcinoma LuCaP 145.1 has lost its epithelial property due to its stemness, and has a lower specific gravity (ρ = 1.035) (39). The tumor cells siphoned off the gradient are washed in HBSS, and checked for purity by RT-PCR analysis for human hB2M and mouse mB2M (19, 39). The cells are plated on MEF in culture media and ROCK inhibitor (10). The media is changed the next day to remove debris and non-adhered cells. After plating on MEF, a starter culture is established. At 60%–70% confluency, the cells are trypsinized and seeded on a new plate of MEF as irradiated MEF do not survive trypsinization. A portion of the LuCaP cells are frozen in 10% DMSO/50% FBS in plastic straws (1/4 cc, γ-irradiated, MAI Animal Health). The sealed straws are quickly chilled to −10°C, then gradually cooled from −10°C to −30°C at 1°C/min (Bio-Cool, SP Scientific). The straws are placed in liquid N_2_. To restart a culture, a single straw of cells is plated on MEF. For example, in the LuCaP 70CR culture described above, small clusters of epithelioid cells were detectable at d3 (62). These individual small colonies expanded such that by d8, large proliferating colonies were evident. The epithelioid appearance of these cells distinguished them from the underlying mouse feeder fibroblasts. This result demonstrated that *in vivo*-passaged LuCaP cells could be frozen for long-term storage and thawed for continuous culture with MEF. The thawed LuCaP 70CR cells survived cloning and multiple passages in the course of over 2 months in that experiment (62).

To prepare MEF culture (113), mouse embryos are removed from pregnant mice at E13.5. The head, heart, and liver are removed. The dissected fetal bodies in phosphate buffered saline (PBS) are passed through an 18-gauge syringe followed by a 23/25-gauge syringe. The dissociated cells are rinsed in DMEM-based MEF media, and seeded to culture dishes. At confluence after 3–4 d, the fibroblasts are passaged and frozen for storage as stocks. The cells are thawed, and approximately 10 culture plates of confluent MEF are resuspended in 5 mL of media for irradiation at 3,000 rad for ~5 min. Approximately 250-μL aliquots of the cell suspension are frozen in individual plastic straws. For use, the irradiated MEF are placed in plates pre-coated with 0.1% gelatin in PBS overnight.

### 2.10 Cell sorting

Both stromal cells of NP and CP can be isolated for downstream work (9). Appropriate excess tissue specimens of resected glands are minced and digested by collagenase, and the resultant single cells are banded on Percoll gradients. Both stromal and epithelial cells are harvested. Dye-conjugated CD49a is used to sort NP stromal cells and CD90 to sort CP stromal cells from tumor samples by AutoMACS (Miltenyi) for culture in 10% FBS-supplemented media (114). The purity of sorted CP stromal cells is checked by RT-PCR for PENK, which should be negative. A positive signal indicates co-purified NP stromal cells (which express low CD90 (12)). The PENK primers are as follows: PENK-5 cagggcccgatatCGCGTCAACTCCATGGCGCGGTTCC, PENK-3 gctgaggatccATTAAAATCTCATAAATCCTCCGTATCTTTTTTC (with the lowercase sequences designed for expression vector cloning (62)). Transcriptomics (55) and proteomics (115) analyses have shown that the cultured stromal cells maintained their overall gene expression so that their functionality can be tested, as in co-culture with EC cells (because of institutional restrictions, ES cells are not available for experimental studies), prostate cancer cells, or any other cell types. Like fibroblasts, stromal cells can be passaged multiple times.

### 2.11 *In vitro* culture of cancer cells and luminal cells on MEF

Since LuCaP xenograft cells can be maintained with MEF, it is possible that cells sorted from resected human tumors could be established *in vitro* directly without employing the xenograft route. To test this possibility, samples of metastatic tumors procured from donor autopsies could be first used in parallel with routine mouse implantation. For sorting after tissue digestion and gradient separation, a number of suitable dye-conjugated antibodies can be used: CD26, CD57, AGR2, and CD106b (11, 13). Similarly, attempts can be made to culture on MEF cancer cells sorted from G3, G4, and G5 primary tumor samples (8). If successful, co-cultures of stromal and cancer cells can be studied for their cell–cell interaction.

**Figure 11:**
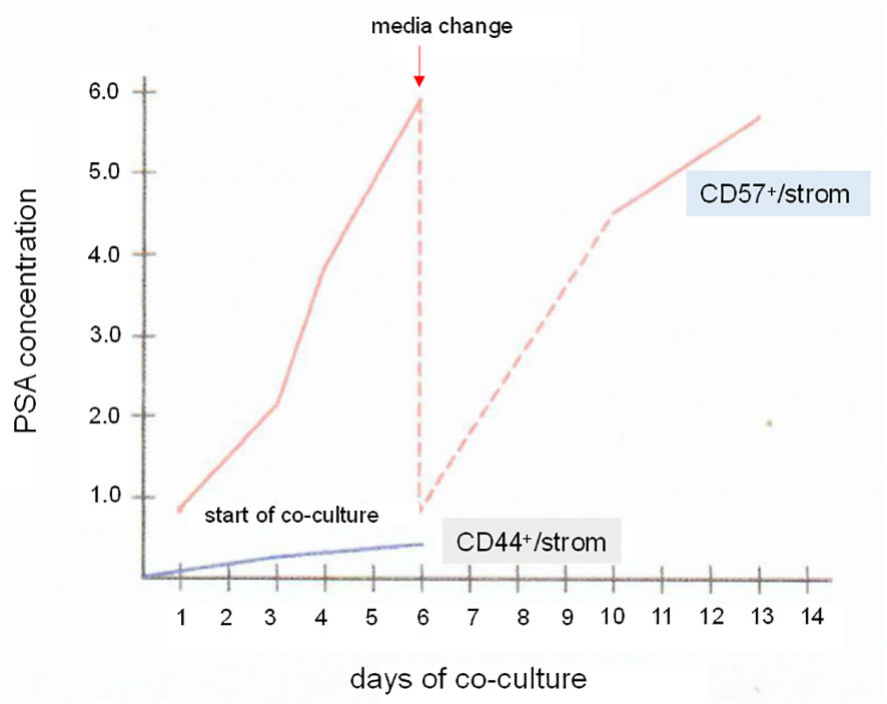
Luminal cells *in vitro*. The plot shows PSA synthesis by CD57 luminal cells in culture with CD49a stromal cells. Samples of the culture media were assayed by PSA ELISA. The PSA level increased over a span of 6 d. The media was changed, and the PSA level again rose afterwards when measured from d10 to d13. For comparison, a co-culture of CD44 basal cells and CD49a showed minimal level of PSA. PSA levels in ng/mL are indicated on the *y*-axis.

A step further, luminal cells could be sorted for culture in MEF in either serum-free (116) or FBS-supplemented media. **Figure 11** shows the influence of stromal cells on PSA synthesis by CD57-sorted luminal cells *in vitro* (60). Isolated luminal cells cease to produce PSA within an hour, and cannot be cultured by themselves (60). In culture with CD49a stromal cells, PSA synthesis was restored. The PSA-synthesizing cells survived a 14-d study period with stromal cells acting as MEF substitute. CD44-sorted basal cells produced no PSA in culture with stromal cells (**Figure 11**). It is then possible that luminal cells on MEF can be propagated and frozen for storage using the cooling protocol described above. If successful, the following studies can be carried out: NP stromal + G3 cancer, NP stromal + G4 cancer, and NP stromal + G5 cancer to determine stromal influence on cancer gene expression, and CP stromal of G3 + luminal, CP stromal of G4 + luminal, and CP stromal of G5 + luminal vs. NP stromal + luminal to determine the effect of stromal signaling on luminal gene expression (cf. **Figure 5D**).

## Author contributions

AL: Writing – original draft.

## References

[1] Gleason DF Mellinger GT. Prediction of prognosis for prostatic adenocarcinoma by combined histological grading and clinical staging. J Urol. (1974) 111:58–64. doi: 10.1016/S0022-5347(17)59889-4 4813554

[2] Minner S Enodien M Sirma H Lübke AM Krohn A Mayer PS. ERG status is unrelated to PSA recurrence in radically operated prostate cancer in the absence of anti-hormonal therapy. Clin Cancer Res. (2011) 17:5878–88. doi: 10.1158/1078-0432.CCR-11-1251 21791629

[3] Shill DK Roobol M Ehdaie B Vickers AJ Carlsson SV. Active surveillance for prosate cancer. Transl Androl Urol. (2021) 10:2809–19. doi: 10.21037/tau PMC8261451 34295763

[4] Egevad L Granfors T Karlberg L Bergh A Stattin P. Prognostic value of the Gleason score in prostate cancer. BJU Int. (2002) 89:538–42. doi: 10.1046/j.1464-410X.2002.02669.x 11942960

[5] Armingol E Officer A Harismendy O Lewis NE. Deciphering cell-cell interactions and communication from gene expression. Nat Rev Genet. (2021) 22:71–88. doi: 10.1038/s41576-020-00292-x 33168968 PMC7649713

[6] Cunha GR. Mesenchymal-epithelial interactions: past, present, and future. Differentiation. (2008) 76:578–86. doi: 10.1111/j.1432-0436.2008.00290.x 18557761

[7] Liu AY Pascal LE Vêncio RZN Vêncio EF. Stromal-epithelial interactions in early neoplasia. Cancer Biomarkers. (2011) 9:141–55. doi: 10.3233/CBM-2011-0174 PMC13015978 22112474

[8] Pascal LE Vêncio RZN Page LS Liebeskind ES Shadle CP Troisch P. Gene expression relationship between prostate cancer cells of Gleason 3, 4 and normal epithelial cells as revealed by cell type-specific transcriptomes. BMC Cancer. (2009) 9:452. doi: 10.1186/1471-2407-9-452 20021671 PMC2809079

[9] Pascal LE Goo YA Vêncio RZN Page LS Chambers AA Liebeskind ES. Gene expression down-regulation in CD90^+^ prostate tumor-associated stromal cells involves potential organ-specific genes. BMC Cancer. (2009) 9:317. doi: 10.1186/1471-2407-9-317 19737398 PMC2745432

[10] Liu AY True LD. Characterization of prostate cell types by CD cell surface molecules. Am J Pathol. (2002) 160:37–43. doi: 10.1016/S0002-9440(10)64346-5 11786396 PMC1867111

[11] Liu AY Roudier MP True LD. Heterogeneity in primary and metastatic prostate cancer as defined by cell surface CD profile. Am J Pathol. (2004) 165:1543–56. doi: 10.1016/S0002-9440(10)63412-8 PMC1618667 15509525

[12] True LD Zhang H Ye M Huang C Nelson PS von Haller PD. CD90/THY1 is overexpressed in prostate cancer-associated fibroblasts and could serve as a cancer biomarker. Mod Pathol. (2010) 23:1346–56. doi: 10.1038/modpathol.2010.122 PMC2948633 20562849

[13] Oudes AJ Campbell DS Sorensen CM Walashek LS True LD Liu AY. Transcriptomes of human prostate cells. BMC Genomics. (2006) 7:92. doi: 10.1186/1471-2164-7-92 16638148 PMC1553448

[14] Liu AY Vêncio RZN Page LS Ho ME Loprieno MA True LD. Bladder expression of CD cell surface antigens and cell-type-specific transcriptomes. Cell Tissue Res. (2012) 348:589–600. doi: 10.1007/s00441-012-1383-y 22427119 PMC3367057

[15] Balzer MS Rohacs T Susztak K. How many cell types are in the kidney and what do they do? Annu Rev Physiol. (2022) 84:507–31. doi: 10.1146/annurev-physiol-052521-121841 PMC9233501 34843404

[16] Da Silva Xavier G. The cells of the islets of Langerhans. J Clin Med. (2018) 7:54. doi: 10.3390/jcm7030054 29534517 PMC5867580

[17] Liu AY True LD LaTray L Ellis WJ Vessella RL Lange PH. Analysis and sorting of prostate cancer cell types by flow cytometry. Prostate. (1999) 40:192–9. 10.1002/(sici)1097-0045(19990801)40:3<192::aid-pros7>3.0.co;2-f 10398281

[18] Pascal LE Vêncio RZN Goo YA Page LS Shadle CP Liu AY. Temporal expression profiling of the effects of secreted factors from prostate stromal cells on embryonal carcinoma stem cells. Prostate. (2009) 69:1353–65. doi: 10.1002/pros.20982 19455603

[19] Borges GT Vêncio EF Quek S Chen A Salvanha DM Vêncio RZN. Conversion of prostate adenocarcinoma to small cell carcinoma-like by reprogramming. J Cell Physiol. (2016) 231:2040–7. doi: 10.1002/jcp.25313 26773436

[20] Vêncio EF Nelson AM Cavanaugh C Ware CB Miller DG Garcia JCO. Reprogramming of prostate cancer-associated stromal cells to embryonic stem-like. Prostate. (2012) 72:1453–63. doi: 10.1002/pros.22497 22314551

[21] Pascal LE Vêncio RZN Vessella RL Ware CB Vêncio EF Denyer G. Lineage relationship of prostate cancer cell types based on gene expression. BMC Med Genomics. (2011) 4:46. doi: 10.1186/1755-8794-4-46 21605402 PMC3113924

[22] Henry NL Hayes DF. Cancer biomarkers. Mol Oncol. (2012) 6:140-146. doi: 10.1016/j.molonc.2012.01.010 PMC5528374 22356776

[23] Espina V Wulfkuhle JD Calvert VS VanMeter A Zhou W Coukos G. Laser-capture microdissection. Nat Protoc. (2006) 1:586–603. doi: 10.1038/nprot.2006.85 17406286

[24] Tomlins SA Rubin MA Chinnaiyan AM. Integrative biology of prostate cancer. Annu Rev Pathol. (2006) 1:243–71. doi: 10.1146/annurev.pathol.1.110304.100047 18039115

[25] True L Coleman I Hawley S Huang C Gifford D Coleman R. A molecular correlate to the Gleason grading system for prostate adenocarcinoma. Proc Natl Acad Sci USA. (2006) 103:10991–6. doi: 10.1073/pnas.0603678103 PMC1544162 16829574

[26] Pascal LE True LD Campbell DS Deutsch EW Risk M Coleman IM. Correlation of mRNA and protein levels: cell type-specific gene expression of cluster designation antigens in the prostate. BMC Genomics. (2008) 9:246. doi: 10.1186/1471-2164-9-246 18501003 PMC2413246

[27] Chen G Ning B Shi T. Single-cell RNA-Seq technologies and related computational data analysis. Front Genet. (2019) 10:317. doi: 10.3389/fgene.2019.00317 31024627 PMC6460256

[28] Pascal LE Deutsch EW Campbell DS Korb M True LD Liu AY. The urologic epithelial stem cell database (UESC) - a web tool for cell type-specific gene expression and immunohistochemistry images of the prostate and bladder. BMC Urol. (2007) 7:19. doi: 10.1186/1471-2490-7-19 18072977 PMC2231381

[29] Fessart D Domblides C Avril T Eriksson LA Begueret H Pineau R. Secretion of protein disulphide isomerase AGR2 confers tumorigenic properties. eLife. (2016) 5:e13887. doi: 10.7554/eLife.13887 27240165 PMC4940162

[30] Maresh EL Mah V Alavi M Horvath S Bagryanova L Liebeskind ES. Differential expression of anterior gradient gene AGR2 in prostate cancer. BMC Cancer. (2010) 10:680. doi: 10.1186/1471-2407-10-680 21144054 PMC3009682

[31] Jach D Cheng Y Prica F Dumartin L Crnogorac-Jurcevic. From development to cancer - an ever-increasing role of AGR2. Am J Cancer Res. (2021) 11:5249–62. PMC8640830 34873459

[32] Sfanos KS De Marzo AM. Prostate cancer and inflammation: the evidence. Histopathology. (2012) 60:199–215. doi: 10.1111/j.1365-2559.2011.04033.x 22212087 PMC4029103

[33] de la Calle CM Shee K Yang H Lonergan PE Nguyen HG. The endoplasmic reticulum stress response in prostate cancer. Nat Rev Urol. (2022) 11:5249–562. doi: 10.1038/S41585-022-00649-3 36168057

[34] Fleischmann A Rocha C Saxer-Sekulic N Zlobec I Sauter G Thalmann GN. High CD10 expression in lymph node metastases from surgically treated prostate cancer independently predicts early death. Virchows Arch. (2011) 458:741–8. doi: 10.1007/s00428-011-1084-z 21538124

[35] Ho ME Quek S True LD Morrissey C Corey E Vessella RL. Prostate cancer cell phenotypes based on AGR2 and CD10 expression. Mod Pathol. (2013) 26:849–59. doi: 10.1038/modpathol.2012.238 PMC3638070 23348903

[36] Dall'Era MA True LD Siegel AF Porter MP Sherertz TM Liu AY. Differential expression of CD10 in prostate cancer and its clinical implication. BMC Urol. (2007) 7:3. doi: 10.1186/1471-2490-7-3 17335564 PMC1829163

[37] Nguyen HM Vessella RL Morrissey C Brown LG Coleman IM Higano CS. LuCaP prostate cancer patient-derived xenografts reflect the molecular heterogeneity of advanced disease and serve as models for evaluating cancer therapeutics. Prostate. (2017) 77:654–71. doi: 10.1002/pros.v77.6 PMC5354949 28156002

[38] Guo H Chen H Zhu Q Yu X Rong R Merugu S. A humanized monoclonal antibody targeting secreted anterior gradient 2 effectively inhibits the xenograft tumor growth. Biochem Biophys Res Commun. (2016) 475:57–63. doi: 10.1016/j.bbrc.2016.05.033 27166154

[39] Kanan AD Corey E Vêncio RZN Ishwar A Liu AY. Lineage relationship between prostate adenocarcinoma and small cell carcinoma. BMC Cancer. (2019) 19:518. doi: 10.1186/s12885-019-5680-7 31146720 PMC6543672

[40] Fritsche FR Dahl E Pahl S Burkhardt M Luo J Mayodomo E. Prognostic relevance of AGR2 expression in breast cancer. Clin Cancer Res. (2006) 12:1728–34. doi: 10.1158/1078-0432.CCR-05-2057 16551856

[41] Zhang K Li Y Kong X Lei C Yang H Wang N. AGR2: a secreted protein worthy of attention in diagnosis and treatment of breast cancer. Front Oncol. (2023) 13:1195885. doi: 10.3389/fonc.2023.1195885 37197416 PMC10183570

[42] Ho ME True LD Seiler R Fleischmann A Bagryanova L Kim SR. Bladder cancer cells secrete while normal bladder cells express but do not secrete AGR2. Oncotarget. (2016) 7:15747. doi: 10.18632/oncotarget.v7i13 26894971 PMC4941274

[43] Dall’Era MA Oudes A Martin DB Liu AY. Identification of HSP27 and HSP70 as CD10 binding proteins in prostate cancer cells. Prostate. (2007) 67:714–21. doi: 10.1002/pros.20558 17342744

[44] Quek S Ho ME Loprieno MA Ellis WJ Elliott N Liu AY. A multiplex assay to measure RNA transcripts of prostate cancer in urine. PloS One. (2012) 9:e45656. doi: 10.1371/journal.pone.0045656 PMC3447789 23029164

[45] Shi T Quek S Gao Y Nicora CD Nie S Fillmore TL. Multiplexed targeted mass spectrometry assays for prostate cancer-associated urinary proteins. Oncotarget. (2017) 8:101887–98. doi: 10.18632/oncotarget.v8i60 PMC5731921 29254211

[46] Quek S Wong OM Chen A Borges GT Ellis WJ Salvanha DM. Processing of voided urine for prostate cancer RNA biomarker analysis. Prostate. (2015) 75:1886–95. doi: 10.1002/pros.23066 26306723

[47] Ring KL Tong LM Balestra ME Javier R Andrews-Zwilling Y Li G. Direct reprogramming of mouse and human fibroblasts into multipotent neural stem cells with a single factor. Cell Stem Cell. (2012) 11:100–9. doi: 10.1016/j.stem.2012.05.018 PMC3399516 22683203

[48] Hepburn AC Steele RE Veeratterapillay R Wilson L Kounatidou EE Barnard A. The induction of core pluripotency master regulators in cancers defines poor clinical outcomes and treatment resistance. Oncogene. (2019) 38:4412–24. doi: 10.1038/s41388-019-0712-y PMC6546609 30742096

[49] Aggarwal R Huang J Alumkal JJ Zhang L Feng FY Thomas GV. Clinical and genomic characterization of treatment-emergent small-cell neuroendocrine prostate cancer: a multi-institutional prospective study. J Clin Oncol. (2018) 36:2492–503. doi: 10.1200/JCO.2017.77.6880 PMC6366813 29985747

[50] Gettinger S Choi J Hastings K Truini A Datar I Sowell R. Impaired HLA class I antigen processing and presentation as a mechanism of acquired resistance to immune checkpoint inhibitors in lung cancer. Cancer Discovery. (2017) 7:1420–35. doi: 10.1158/2159-8290.CD-17-0593 PMC5718941 29025772

[51] Choi K Yu J Smuga-Otto K Salvagiotto G Rehrauer W Vodyanik M. Hematopoietic and endothelial differentiation of human induced pluripotent stem cells. Stem Cells. (2009) 27:559–67. doi: 10.1002/stem.v27:3 PMC2931800 19259936

[52] Al Abbar A Ngai SC Nograles N Alhaji SY Abdullah S. Induced pluripotent stem cells: reprogramming platforms and applications in cell replacement therapy. Biores Open Access. (2020) 9:121–36. doi: 10.1089/biores.2019.0046 PMC7194323 32368414

[53] Cunha GR Ricke W Thomson A Marker PC Risbridger G Hayward SW. Hormonal, cellular, and molecular regulation of normal and neoplastic prostatic development. J Steroid Biochem Mol Biol. (2004) 92:221–36. doi: 10.1016/j.jsbmb.2004.10.017 15663986

[54] Damjanov I Horvat B Gibas Z. Retinoic acid-induced differentiation of the developmentally pluripotent human germ cell tumor-derived cell line, NCCIT. Lab Invest. (1993) 68:220–32. 7680083

[55] Goo YA Goodlett DR Pascal LE Worthington KD Vessella RL True LD. Stromal mesenchyme cell genes of the human prostate and bladder. BMC Urol. (2005) 5:17. doi: 10.1186/1471-2490-5-17 16343351 PMC1327674

[56] Vaalasti A Linnoila I Hervonen A. Immunohistochemical demonstration of VIP, [Met5]- and [Leu5]-enkephalin immunoreactive nerve fibres in the human prostate and seminal vesicles. Histochemistry. (1980) 66:89–98. doi: 10.1007/BF00493249 6993434

[57] Rosen H Krichevsky A Polakiewicz RD Benzakine S Bar-Shavit Z. Developmental regulation of proenkephalin gene expression in osteoblasts. Mol Endocrinol. (1995) 9:1621–31. doi: 10.1210/mend.9.11.8584038 8584038

[58] Pascal LE Ai J Vêncio RZN Vêncio EF Zhou Y Page LS. Differential inductive signaling of CD90^+^ prostate cancer-associated fibroblasts compared to normal tissue stromal mesenchyme cells. Cancer Microenviron. (2011) 4:51–9. doi: 10.1007/s12307-010-0061-4 PMC3047627 21505567

[59] de Moraes RP Pimenta R Mori FNC Dos Santos GA Viana NI Guimarães VR. Tissue expression of MMP-9, TIMP-1, RECK, and miR338-3p in prostate gland: can it predict cancer? Mol Biol Res Commun. (2021) 10:149–56. doi: 10.22099/mbrc.2021.40912.1646 PMC8798274 35097136

[60] Liu AY True LD LaTray L Nelson PS Ellis WJ Vessella RL. Cell-cell interaction in prostate gene regulation and cytodifferentiation. Proc Natl Acad Sci USA. (1997) 94:10705–10. doi: 10.1073/pnas.94.20.10705 PMC23453 9380699

[61] Vêncio EF Pascal LE Page LS Denyer G Wang AJ Ruohola-Baker H. Embryonal carcinoma cell induction of miRNA and mRNA changes in co-cultured prostate stromal fibromuscular cells. J Cell Physiol. (2011) 226:1479–88. doi: 10.1002/jcp22464 PMC3968429 20945389

[62] Liu AY. The opposing action of stromal cell proenkephalin and stem cell transcription factors in prostate cancer differentiation. BMC Cancer. (2021) 21:1335. doi: 10.1186/s12885-021-09090-y 34911496 PMC8675470

[63] Joung J Ma S Tay T Geiger-Schuller KR Kirchgatterer PC Verdine VK. A transcription factor atlas of directed differentiation. Cell. (2023) 186:209–29. doi: 10.1016/j.cell.2022.11.026 PMC10344468 36608654

[64] Labreque MP Coleman IM Brown LG True LD Kollath L Lakely B. Molecular profiling stratifies diverse phenotypes of treatment-refractory metastatic castration-resistant prostate cancer. J Clin Invest. (2019) 130:4492–505. doi: 10.1172/JCI128212 PMC6763249 31361600

[65] Wayner EA Quek S Ahmad R Ho ME Loprieno MA Zhou Y. Development of an ELISA to detect the secreted prostate cancer biomarker AGR2 in voided urine. Prostate. (2012) 72:1023–34. doi: 10.1002/pros.21508 22072305

[66] Tewari A ed. Prostate cancer: a comprehensive perspective. Springer Verlag London (2013). doi: 10.1007/978-1-4471-2864-9

[67] Chung C Abboud K. Targeting the androgen receptor signaling pathway in advanced prostate cancer. Am J Health Syst Pharm. (2022) 79:1224–35. doi: 10.1093/ajhp/zxac105 35390118

[68] Robinson D Van Allen EM Wu YM Schultz N Lonigro RJ Mosquera JM. Integrative clinical genomics of advanced prostate cancer. Cell. (2015) 161:1215–28. doi: 10.1016/j.cell.2015.05.001 PMC4484602 26000489

[69] Seiler R von Gunten M Thalmann GN Fleischmann A. High CD10 expression predicts favorable outcome in surgically treated lymph node-positive bladder cancer patients. Hum Pathol. (2012) 43:269–75. doi: 10.1016/j.humpath.2011.04.030 21835428

[70] Alavi M Mah V Maresh EL Bagryanova L Horvath S Chia D. High expression of AGR2 in lung cancer is predictive of poor survival. BMC Cancer. (2015) 15:655. doi: 10.1186/s12885-015-1658-2 26445321 PMC4596313

[71] Kristiansen G Schlüns K Yongwei Y Dietel M Petersen I. CD10 expression in non-small cell lung cancer. Anal Cell Pathol. (2002) 24:41–6. doi: 10.1155/2002/781580 PMC4617995 12122282

[72] Deutsch EW. The peptideatlas project. Methods Mol Biol. (2010) 604:285–96. doi: 10.1007/978-1-60761-449-9_19 PMC3076596 20013378

[73] Nagaraj N Mann MJ. Quantitative analysis of the intra- and inter-individual variability of the normal urinary proteome. J Proteome Res. (2011) 10:637–45. doi: 10.1021/pr100835s 21126025

[74] Shi T Gao Y Quek S Fillmore TL Nicora CD Su D. A highly sensitive targeted mass spectrometric assay for quantification of low-abundance AGR2 in human urine and serum. J Proteome Res. (2014) 13:875–82. doi: 10.1021/pr400912c PMC3975687 24251762

[75] Hu R Huffman KE Chu K Zhang Y Minna JD Yu Y. Quantitative secretomic analysis identifies extracellular protein factors that modulate the metastatic phenotype of non-small cell lung cancer. J Proteome Res. (2016) 15:477–86. doi: 10.1021/acs.jproteome.5b00819 PMC5001500 26736068

[76] Hingorani SR Wang L Multani AS Combs C Deramaudt TB Hruban RH. Trp53* ^R172H^ * and Kras* ^G12D^ * cooperate to promote chromosomal instability and widely metastatic pancreatic ductal adenocarcinoma in mice. Cancer Cell. (2005) 7:469–83. doi: 10.1016/j.ccr.2005.04.023 15894267

[77] Liu AY Kanan AD Radon TP Shah S Weeks ME Foster JM. AGR2, a unique tumor-associated antigen, is a promising candidate for antibody targeting. Oncotarget. (2019) 10:4276–89. doi: 10.18632/oncotarget.v10i42 PMC6611513 31303962

[78] Kumar A White TA MacKenzie AP Clegg N Lee C Dumpit RF. Exome sequencing identifies a spectrum of mutation frequencies in advanced and lethal prostate cancers. Proc Natl Acad Sci USA. (2011) 108:17087–92. doi: 10.1073/pnas.1108745108 PMC3193229 21949389

[79] Tai W Mahato R Cheng K. The role of HER2 in cancer therapy and targeted drug delivery. J Control Release. (2010) 146:264–75. doi: 10.1016/j.jconrel.2010.04.009 PMC2918695 20385184

[80] Bournazos C Chow SK Abboud N Casadevall A Ravetch JV. Human IgG Fc domain engineering enhances antitoxin neutralizing antibody activity. J Clin Invest. (2014) 124:725–9. doi: 10.1172/JCI72676 PMC3904629 24401277

[81] Derer S Beurskens FJ Rosner T Peipp M Valerius T. Complement in antibody-based tumor therapy. Crit Rev Immunol. (2014) 34:199–214. doi: 10.1615/CritRevImmunol.v34.i3 24941073

[82] Vidarsson G Dekkers G Rispens T. IgG subclasses and allotypes: from structure to effector functions. Front Immunol. (2014) 5:520. doi: 10.3389/fimmu.2014.00520 25368619 PMC4202688

[83] Liu AY Robinson RR Murray ED Ledbetter JA Hellström I Hellström KE. Production of a mouse:human chimeric monoclonal antibody to CD20 with potent F_c_-dependent biologic activity. J Immunol. (1987) 139:3521–6. doi: 10.4049/jimmunol.139.10.3521 3119711

[84] Liu AY Robinson RR Hellström KE Murray ED Chang CP Hellström I. Chimeric mouse:human IgG1 antibody that can mediate lysis of cancer cells. Proc Natl Acad Sci USA. (1987) 84:3439–43. doi: 10.1073/pnas.84.10.3439 PMC304886 3106970

[85] Baxevanis CN Papamichail M Perez SA. Prostate cancer vaccines: the long road to clinical application. Cancer Immunol Immunother. (2015) 64:401–8. doi: 10.1007/s00262-015-1667-7 PMC11029136 25690791

[86] Bargão Santos P Patel HR. Prostate stem cell antigen – novel biomarker and therapeutic target? Expert Rev Anticancer Ther. (2014) 14:5–7. doi: 10.1586/14737140.2014.870481 24320701

[87] Goswami S Aparicio A Subudhi SK. Immune checkpoint therapies in prostate cancer. Cancer J. (2016) 22:117–20. doi: 10.1097/PPO.0000000000000176 PMC4847149 27111907

[88] Petrylak DP Kantoff P Vogelzang NJ Mega A Fleming MT Stephenson JJ. Phase 1 study of PSMA ADC, an antibody-drug conjugate targeting prostate-specific membrane antigen, in chemotherapy-refractory prostate cancer. Prostate. (2019) 79:604–13. doi: 10.1002/pros.23765 30663074

[89] Barinka C Šácha P Sklenář J Man P Bezouška K Slusher BS. Identification of the *N*-glycolysis sites in glutamate carboxypeptidase II necessary for proteolytic activity. Protein Sci. (2004) 13:1627–35. doi: 10.1110/ps.04622104 PMC2279971 15152093

[90] DiPippo VA Olson WC Nguyen HM Brown LG Vessella RL Corey E. Efficacy studies of an antibody-drug PSMA-ADC in patient-derived prostate cancer xenografts. Prostate. (2015) 75:303–13. doi: 10.1002/pros.22916 25327986

[91] Magee MS Snook AE. Challenges to chimeric antigen receptor (CAR)-T cell therapy for cancer. Discovery Med. (2014) 18:265–71. PMC12321268 25425467

[92] Lee HJ Hong CY Kim MH Lee YK Nguyen-Pham TN Park BC. *In vitro* induction of anterior gradient-2-specific cytotoxic T lymphocytes by dendritic cells transduced with recombinant adenoviruses as a potential therapy for colorectal cancer. Exp Mol Med. (2012) 44:60–7. doi: 10.3858/emm.2012.44.1.006 PMC3277899 22089087

[93] Vitello EA Quek S Kincaid H Fuchs T Crichton DJ Troisch P. Cancer-secreted AGR2 induces programmed cell death of normal cells. Oncotarget. (2016) 7:49425–34. doi: 10.18632/oncotarget.v7i31 PMC5226518 27283903

[94] Tereshina MB Ivanova A Eroshkin FM Korotkova DD Nesterenko AM Bayramov AV. Agr2-interacting Prod1-like protein Tfp4 from *Xenopus laevis* is necessary for early forebrain and eye development as well as for the tadpole appendage regeneration. Genesis. (2019) 57:323293. doi: 10.1002/dvg.23293 30912273

[95] Enane FO Sauntharararjah Y Korc M. Differentiation therapy and the mechanisms that terminate cancer cell proliferation without harming normal cells. Cell Death Dis. (2018) 9:912. doi: 10.1038/s41419-018-0919-9 30190481 PMC6127320

[96] de Thé H. Differentiation therapy revisited. Nat Rev Cancer. (2018) 18:117–27. doi: 10.1038/nrc.2017.103 29192213

[97] Dela Cruz F Matushansky I. Solid tumor differentiation therapy – is it possible? Oncotarget. (2012) 3:559–67. doi: 10.18632/oncotarget.v3i5 PMC3388185 22643847

[98] Spengler G Csonka Á Molnár J Amaral L. The anticancer activity of the old neuroleptic phenothiazine-type drug thioridazine. Anticancer Res. (2016) 36:5701–6. doi: 10.21873/anticanres 27793891

[99] Loehr AR Pierpont TM Gelsleichter E Galang AMD Fernandez IR Moore ES. Targeting cancer stem cells with differentiation agents as an alternative to genotoxic chemotherapy for the treatment of malignant testicular germ cell tumors. Cancers. (2021) 13:2045. doi: 10.3390/cancers13092045 33922599 PMC8122873

[100] Lee SI Roney MSI Park JH Baek J Park J Kim SK. Dopamine receptor antagonists induce differentiation of PC-3 human prostate cancer cell-derived cancer stem cell-like cells. Prostate. (2019) 79:720–31. doi: 10.1002/pros.23779 30816566

[101] Fong D Christensen CT Chan MM. Targeting cancer stem cells with repurposed drugs to improve current therapies. Recent Pat Anticancer Drug Discovery. (2021) 16:136–60. doi: 10.2174/1574892816666210208232251 33563159

[102] Rosas-Cruz A Salinas-Jazmín N Velasco-Velázquez M. Dopamine receptors in cancer: are they valid therapeutic targets. Technol Cancer Res Treat. (2021) 20:15330338211027913. doi: 10.1177/15330338211027913 34212819 PMC8255587

[103] Ben-Porath I Thomson MW Carey VJ Ge R Bell GW Regev A. An embryonic stem cell-like gene expression signature in poorly differentiated aggressive human tumors. Nat Genet. (2008) 40:499–507. doi: 10.1038/ng.127 18443585 PMC2912221

[104] Jiang WQ Chang AC Satoh M Furuichi Y Tam PP Reddel RR. The distribution of stanniocalcin 1 protein in fetal mouse tissues suggests role in bone and muscle development. J Endocrinol. (2000) 165:457–66. doi: 10.1677/joe.0.1650457 10810309

[105] Strasko SE Wagner GF. Stanniocalcin gene expression during mouse urogenital development: a possible role in mesenchymal-epithelial signaling. Dev Dyn. (2001) 220:49–59. doi: 10.1002/1097-0177(2000)9999:9999<::AID-DVDY1086>3.0.CO;2-5 11146507

[106] Ellis WJ Vessella RL Buhler KR Bladou F True LD Bigler SA. Characterization of a novel androgen-sensitive, prostate-specific antigen-producing prostatic carcinoma xenograft: LuCaP 23. Clin Cancer Res. (1996) 2:1039–48. 9816265

[107] Namekawa T Ikeda K Horie-Inoue K Inoue S. Application of prostate cancer models for preclinical study: advantages and limitations of cell lines, patient-derived xenografts, and three-dimensional culture of patient-derived cells. Cells. (2019) 8:74. doi: 10.3390/cells8010074 30669516 PMC6357050

[108] Klein KA Reiter RE Redula J Moradi H Zhu XL Brothman AR. Progression of metastatic human prostate cancer to androgen independence in immunodeficient SCID mice. Nat Med. (1997) 3:402–8. doi: 10.1038/nm0497-402 9095173

[109] Young SR Saar M Santos J Nguyen HM Vessella RL Peehl DM. Establishment and serial passage of cell cultures derived from LuCaP xenografts. Prostate. (2013) 73:1251–62. doi: 10.1002/pros.22610 PMC3720815 23740600

[110] Saar M Zhao H Nolley R Young SR Coleman I Nelson PS. Spheroid culture of LuCaP 147 as an authentic preclinical model of prostate cancer subtype with SPOP mutation and hypermutator phenotype. Cancer Lett. (2014) 351:272–80. doi: 10.1016/j.canlet.2014.06.014 PMC4112013 24998678

[111] Valta MP Zhao H Saar M Tuomela J Nolley R Linxweiler J. Spheroid culture of LuCaP 136 patient-derived xenograft enables versatile preclinical models of prostate cancer. Clin Exp Metastasis. (2016) 33:325–37. doi: 10.1007/s10585-016-9781-2 PMC12997442 26873136

[112] True LD Buhler K Quinn J Williams E Nelson PS Clegg N. A neuroendocrine/small cell carcinoma xenograft – LuCaP 49. Am J Pathol. (2002) 161:705–15. doi: 10.1016/S0002-9440(10)64226-5 PMC1850754 12163395

[113] Abbondanzo SJ Gadi I Stewart CL. Derivation of embryonic stem cell lines. Methods Enzymol. (1993) 225:803–23. doi: 10.1016/0076-6879(93)25052-4 8231888

[114] Liu AY LaTray L van den Engh G. Changes in cell surface molecules associated with in *vitro* culture of prostatic stromal cells. Prostate. (2000) 44:303–12. doi: 10.1002/(ISSN)1097-0045 10951495

[115] Goo YA Liu AY Ryu S Shaffer SA Malmström L Page L. Identification of secreted glycoproteins of human prostate and bladder stromal cells by comparative quantitative proteomics. Prostate. (2008) 69:49–61. doi: 10.1002/pros.20853 PMC4281891 18792917

[116] Liu AY Peehl DM. Characterization of cultured human prostatic epithelial cells by cluster designation antigen expression. Cell Tissue Res. (2001) 305:389–97. doi: 10.1007/s004410100419 11572092

